# Multi-ingredient pre-workout supplements, safety implications, and performance outcomes: a brief review

**DOI:** 10.1186/s12970-018-0247-6

**Published:** 2018-08-08

**Authors:** Patrick S. Harty, Hannah A. Zabriskie, Jacob L. Erickson, Paul E. Molling, Chad M. Kerksick, Andrew R. Jagim

**Affiliations:** 1 School of Health Sciences, Department of Exercise Science, Lindenwood University, St. Charles, MO 63301 USA; 2 Mayo Clinic Health Systems, Onalaska, WI 54650 USA

**Keywords:** Ergogenic aid, Supplement, Pre-workout, Strength, Power, Energy, Resistance-training

## Abstract

In recent years, a new class of dietary supplements called multi-ingredient pre-workout supplements (MIPS) has increased in popularity. These supplements are intended to be taken prior to exercise and typically contain a blend of ingredients such as caffeine, creatine, beta-alanine, amino acids, and nitric oxide agents, the combination of which may elicit a synergistic effect on acute exercise performance and subsequent training adaptations compared to single ingredients alone. Therefore, the purpose of this article was to review the theoretical rationale and available scientific evidence assessing the potential ergogenic value of acute and chronic ingestion of MIPS, to address potential safety concerns surrounding MIPS supplementation, and to highlight potential areas for future research. Though direct comparisons between formulations of MIPS or between a MIPS and a single ingredient are challenging and often impossible due to the widespread use of “proprietary blends” that do not disclose specific amounts of ingredients in a given formulation, a substantial body of evidence suggests that the acute pre-exercise consumption of MIPS may positively influence muscular endurance and subjective mood, though mixed results have been reported regarding the acute effect of MIPS on force and power production. The chronic consumption of MIPS in conjunction with a periodized resistance training program appears to augment beneficial changes in body composition through increased lean mass accretion. However, the impact of long-term MIPS supplementation on force production, muscular endurance, aerobic performance, and subjective measures is less clear. MIPS ingestion appears to be relatively safe, though most studies that have assessed the safety of MIPS are relatively short (less than eight weeks) and thus more information is needed regarding the safety of long-term supplementation. As with any dietary supplement, the use of MIPS carries implications for the athlete, as many formulations may intentionally contain banned substances as ingredients or unintentionally as contaminants. We suggest that athletes thoroughly investigate the ingredients present in a given MIPS prior to consumption. In conclusion, it appears that multi-ingredient pre-workout supplements have promise as an ergogenic aid for active individuals, though further information is required regarding long-term efficacy and safety in a wider variety of populations.

## Background

The use of nutritional supplements to improve performance and augment training adaptations is becoming increasingly prevalent in today’s world of sports and fitness [1, 2]. A new class of dietary supplements known as multi-ingredient pre-workout supplements (MIPS) has garnered interest from athletes, fitness enthusiasts, and researchers alike. MIPS products typically contain a blend of ingredients such as caffeine, branched-chain amino acids, nitrates, creatine, β-alanine, and other ingredients that are purported to improve acute exercise performance, potentially leading to augmented training adaptations following continued use [3–5]. These ingredients are often displayed on the supplement facts label in the form of a “proprietary blend,” with specific amounts sometimes not disclosed to the consumer. Though United States Food and Drug Administration (FDA) regulations require that all dietary ingredients included in proprietary blends are listed in descending order of predominance by weight, such labeling practices make it challenging to determine whether a supplement contains sufficient amounts of key ergogenic ingredients. For example, one ingredient listed at the beginning of a proprietary blend may be over-represented in the blend, while other ingredients may be included at quantities far below the threshold of efficacy. Because many of the common ingredients used in MIPS formulations act on different physiological mechanisms, many researchers have speculated whether certain combinations of these ingredients may confer a synergistic effect on the overall efficacy of a given formulation [5]. However, as the majority of MIPS that have been investigated in the literature do not list ingredients beyond a proprietary blend, it becomes difficult to make direct comparisons between the effects of a given MIPS and the equivalent amount of an individual ingredient or from one product to another. This problem is evident in the literature, as nearly all studies that have investigated MIPS have not compared the effects of these supplements against those of a recognized primary ingredient such as creatine, caffeine, or β-alanine. However, one notable exception to this trend is a recent investigation by Lane and Byrd [6], which compared the effects of a caffeine-containing MIPS against a caffeine-matched placebo and an inert placebo alone. The researchers found that bench press peak velocity was significantly improved over inert placebo after ingestion of both MIPS or caffeine, though bench press mean velocity was only significantly improved after consumption of MIPS. Furthermore, both the MIPS and the caffeine condition were found to have no demonstrable effect on blood lactate levels, vertical jump performance, and repeat Wingate anaerobic cycling performance, suggesting the presence of similarities between the effects of the MIPS in question and caffeine alone. Conversely, Shields et al. [7] compared between the effects of caffeine and a caffeine-free MIPS formulation in a pilot study and reported that only subjects who consumed caffeine exhibited decreased calmness, a common side effect of the substance. Future research should utilize similar study designs to allow for direct comparison between the effects of a MIPS and one or more of its constituent ingredients, as the results of such investigations allow for one to determine the uniqueness and efficacy of proprietary blends. Despite the difficulties in comparing between MIPS and other ergogenic substances, many off-the shelf MIPS formulations have been found to significantly improve acute exercise performance and enhance training adaptations over time when combined with a structured training program. Due to their relative novelty, it is important to examine the efficacy and safety following both acute and chronic ingestion of these products before providing recommendations to the consumer. Unfortunately, many of the published works on this topic do not include clearly delineated primary outcome measures and instead focus on all statistically significant outcomes to bolster their strength and conclusions. Such an approach may skew the conclusions of the reader, as only the positive aspects of a given supplement are emphasized. As the purpose of this article was to review the entire body of scientific evidence assessing the potential ergogenic value of acute and chronic ingestion of MIPS, no outcome measure from any relevant study was excluded from our analysis, allowing for a more complete and balanced overview of the literature. In addition, this review addresses the potential safety concerns surrounding MIPS supplementation and potential areas for future research. This was accomplished by the completion of a thorough review of the published literature investigating the effects of MIPS ingestion on exercise performance and potential for the enhancement of training adaptations over time.

## Methods

Ending on May 2, 2018, PubMed, Ebsco Host, Medline, and Google Scholar databases were searched for published literature using but not limited to the following keywords: multi-ingredient pre-workout supplement, MIPS, pre-workout, multi-ingredient performance supplement. To facilitate interpretation of published results, the authors of this review calculated effect sizes when not provided by included texts. Standardized mean difference (Cohen’s *d*) was calculated for each statistically significant (*p* < 0.05) outcome measure using the following formula: *d* = (Improvement_MIPS_ – Improvement_Control_)/ SD_pooled_ [8]. Effects were considered very weak if they were less than 0.2, small if between 0.2–0.5, moderate if between 0.5–0.8, and strong if greater than 0.8. In cases where Cohen’s *d* could not be calculated due to unreported sample size or standard deviation, Relative Effects (RE) were calculated as follows: RE = [(Post_MIPS_/Pre_MIPS_)*100 / (Post_Control_/Pre_Control_)*100]*100 according to the methods employed by Trexler et al. [9] in a recent position stand. A relative effect greater than 100 indicates MIPS increased the outcome measure, while a value less than 100 indicates MIPS decreased the outcome measure. However, there were still studies in which *d* and RE were impossible to calculate due to missing data or data being presented in a figure without data labels. In these instances, the effect size is listed as NA. All effect measurements, the statistical significance of each effect, and the strength of each effect are presented in parentheses throughout the text. Because direct comparisons between different formulations of MIPS remain challenging due to wide variations in ingredients and dosages, summary tables have been developed that outline: dosing, MIPS ingredients listed in descending order of predominance by weight, exercise interventions, effect size, and other relevant information to present a comprehensive overview of each study.

## Ingredients

As the ergogenic effects of many of the primary MIPS ingredients have been examined on an individual basis and reviewed elsewhere, the following section will briefly outline the mechanism of action, common dosing protocols, and ergogenic potential of common MIPS ingredients. For the purposes of this review, the term MIPS will be confined to multi-ingredient pre-workout supplements and is not to be confused with energy drinks, energy shots, or commercial sport drinks (previously reviewed in Ref. [10]), which, despite having some overlapping ingredients such as caffeine, tend to differ in specific ingredient profiles and purported benefits.

### Caffeine

Caffeine (Reviewed extensively in Refs. [11–13]) appears to be the primary ingredient responsible for several of the acute ergogenic effects of MIPS, as it is rapidly absorbed and typically peaks in the bloodstream within 60 min of ingestion [5, 11]. Caffeine acts as an adenosine receptor antagonist [14] and has been shown to acutely improve cognition as well as performance during endurance, power, and resistance exercise when consumed in dosages between 3 and 6 mg/kg bodyweight [11]. MIPS formulations that contain at least 300 mg caffeine per serving will fall within the acceptable dosing range for most individuals.

### Amino acids and amino-containing compounds

Taurine is an amino-containing sulfonic acid that has been reported to have antioxidant, metabolic, and ergogenic effects [15]. While chronic consumption of the substance may improve time-to-exhaustion during endurance exercise, acute ingestion of 1.5 g taurine as part of a MIPS has been shown to improve muscular endurance during resistance exercise [16]. Branched-chain amino acids (BCAAs) are often added to MIPS formulations with the intent of boosting rates of muscle protein synthesis, minimizing protein breakdown, and reducing exercise-induced muscle damage [17]. While BCAAs have been theorized to ameliorate fatigue, they do not appear to significantly enhance exercise performance or stimulate muscle protein synthesis [17, 18].

### Nitric oxide agents

Nitric oxide (NO) is a vital signaling molecule that has been shown to increase blood flow to active muscles, which in theory may result in increased exercise performance [19–21]. Several common components of MIPS (i.e. arginine, citrulline) are purported to increase levels of nitric oxide and thus improve performance via increased blood flow during exercise, with equivocal performance outcomes reported in the literature [22]. Dietary nitrate (Reviewed in Refs. [19–22] appears to enhance acute endurance and high-intensity exercise performance when consumed in doses of 300 mg or higher [23]. Inorganic nitrate (NO_3_^−^) is a compound found in beetroot juice and sodium nitrate that is reduced to Nitrite (NO_2_^−^) in the oral cavity via enzymatic activity and then to nitric oxide (NO) in the stomach or peripheral tissues under hypoxic conditions [19–21]. L-arginine (Reviewed in Refs. [22, 24]) is an amino acid that is a precursor required for the synthesis of nitric oxide. While some studies have demonstrated ergogenic benefits resulting from oral consumption of L-arginine, the majority of evidence suggests that arginine has limited efficacy in improving blood flow or exercise performance. L-Citrulline (Reviewed in Refs. [22, 25]) is a non-essential amino acid found primarily in watermelon that is converted to L-arginine and thus promotes NO synthesis. L-Citrulline supplementation (often combined with malate, an intermediate in the citric acid cycle) has been shown to increase vasodilation and improve exercise performance when consumed chronically in doses of 6-8 g per day [22, 25]. However, the doses found in MIPS are generally far lower than those that have shown ergogenic effects.

### Creatine

Creatine (Reviewed in Ref [26]) is a naturally-occurring amino acid found in the muscle of various animals [27]. Creatine supplementation is safe and has been consistently demonstrated to increase intramuscular phosphocreatine levels by 30% after supplementing with roughly 5 g (0.03 g/kg/dose) per day following a loading period of 20 g/day (0.3 g/kg/day). This regimen has gone on to positively impact high-intensity exercise performance when chronically consumed in doses equal to roughly 3-5 g per day following a loading period of 20 g/day [26]. While post-workout creatine consumption appears to be superior to pre-exercise supplementation [28], the regular consumption of at least 3 g of creatine from a MIPS per day for 28 days is likely sufficient to enhance exercise performance and augment training adaptations [26].

### Betaine

Betaine (trimethylglycine) is a naturally-occurring derivative of the amino acid glycine which may improve exercise performance by increasing rates of creatine synthesis, elevating levels of blood nitric oxide, and promoting fluid and thermal homeostasis [29, 30]. Chronic supplementation with 1.25–2.5 g per day of betaine has been demonstrated to enhance repetitions to fatigue and total volume load completed during resistance exercise [29, 30], with improvements in power and force production also reported [31]. However, the dosages used in these investigations are far higher than those found in a typical MIPS.

### Beta-alanine

β-alanine (Reviewed in Ref. [9]) is a common MIPS component and precursor to carnosine, a dipeptide which acts as an intramuscular buffer. The consumption of 4-6 g β-alanine per day over a period of at least 2 weeks has been demonstrated to improve high-intensity exercise performance [9]. Thus, provided that a MIPS contains sufficient amounts of β-alanine, similar ergogenic effects can be expected, if consumed daily to appropriately maintain intramuscular carnosine levels.

## Acute effects of MIPS on performance

Numerous investigations have examined the effect of a single pre-exercise dose of MIPS on performance outcomes. These studies are outlined in Table 1.

**Table 1:** Performance outcomes in acute MIPS studies

Study	Subjects	Design	Supplement Ingredients (Sorted by order listed on Supplement Facts label)	Timing	Performance Testing Protocol	Results (*d* or RE)	Ref #
Hoffman et al. 2008	8 college-aged, resistance-trained males	Randomized, Double-blind, placebo-controlled, crossover design.	BCAAs (5200 mg) Creatine (5000 mg) Essential Amino Acids (4300 mg) Taurine (1500 mg) Glucuronolactone (350 mg) Caffeine (110 mg)	20 min prior to exercise	6 sets of up to 10 repetitions Barbell Back Squat, 75% 1RM	↑ Repetitions during Set 5 (NA)	[16]
Hoffman et al. 2009	12 college-aged male strength and power athletes	Randomized, Double-blind, placebo-controlled, crossover design.	*Prop. Blend: (726 mg)* β-alanine Caffeine (158 mg) L-Leucine B-Phenylethylamine HCl L-Valine L-Isoleucine N-Acetyl-L-Tyrosine Yohimbine 5-hydroxytryptophan	10 min prior to exercise	Choice Reaction Time Test, 20-s WAnT repeated three times	↑ Number of Targets Struck (*d* = 0.53) ↑ Percent of Targets Stuck (*d* = 0.48) ↔ Peak Power ↔ Mean Power	[43]
Bloomer et al. 2010	19 resistance-trained males	Randomized, Double-blind, placebo-controlled, crossover design.	*3 MIPS Investigated, all contained the following:* Amino Acids Creatine Taurine Caffeine (MIPS 3: 234 mg)	30 min prior to exercise	Bench Press Throws at 30% 1RM, 10 sets of BP RTF at 50% 1RM	↔ BP 1RM ↔ RTF ↔Upper Body Power	[35]
Hoffman et al. 2010	19 recreationally-active males and females	Randomized, Double-blind, placebo-controlled design.	Acetyl-L-carnitine (500 mg) L-tyrosine (500 mg) Α-glycerophosphocholine (150 mg) Choline bitartrate (125 mg) Caffeine (60 mg) Phosphatidylserine (50 mg)	10 min prior to exercise	Choice Reaction Time Test before and after the following: WAnT, Maximum Pushup Repetitions Completed in one Minute, Maximum Situp Repetitions Completed in one Minute	↑ Retention of Reaction Time (NA) ↔ Peak Power ↔ Mean Power ↔ Pushups Completed ↔ Sit-ups Completed	[36]
Walsh et al. 2010	15 recreationally-trained males and females	Randomized, Double-blind, placebo-controlled, crossover design.	^a^ *Prop. Blend 1: (7900 mg)* L-Leucine, L-Arginine, L-Glutamine, L-Valine, L-Isoleucine *Prop. Blend 2: (2050 mg)* Taurine, Glucoronolactone, Caffeine β-alanine (2500 mg) Creatine (5000 mg) Vitamin C (7900 mg)	10 min prior to exercise	Treadmill TTE Protocol at 70% VO_2_max	↑ TTE (NA)	[44]
Gonzalez et al. 2011	8 college-aged, resistance-trained males	Randomized, Double-blind, placebo-controlled, crossover design.	^a^ *Prop. Blend 1: (7900 mg)* L-Leucine, L-Arginine, L-Glutamine, L-Valine, L-Isoleucine *Prop. Blend 2: (2050 mg)* Taurine, Glucoronolactone, Caffeine β-alanine (2500 mg) Creatine (5000 mg) Vitamin C (7900 mg)	10 min prior to exercise	4 sets of up to 10 repetitions BP at 80% 1RM	↑ Total Lifting Volume (*d* = 0.30) ↑ BP Peak Power (*d* = 0.53) ↑ BP Mean power (*d* = 0.29)	[38]
Spradley et al. 2012	12 recreationally-trained males	Randomized, Double-blind, placebo-controlled, crossover design.	^b^BCAAs (6000 mg) Creatine (5000 mg) β-alanine (4000 mg) Citrulline malate (1500 mg) Caffeine (300 mg)	20 min prior to exercise	LP and BP RTF Repeated 2 Times, Choice Reaction Time Test Repeated 4 Times, Intermittent Critical Velocity Test Repeated 4 Times	↑ LP RTF (T1: *d* = 0.42; T2: *d* = 0.72) ↑ Choice Reaction Time (Audio Single Step: *d* = − 0.47; 15 s Multi-Directional: *d* = − 0.46; 30s Multi-Directional: *d* = − 0.33) ↔ BP RTF ↔ Critical Velocity ↔ Anaerobic Running Capacity	[37]
Jacobs 2014	19 recreationally-active males and females	Randomized, Double-blind, placebo-controlled, crossover design.	*Prop. Blend 1: (13,000 mg)* Brown rice extract β-alanine Agmatine sulfate Grape seed extract Caffeine	20 min prior to exercise	Maximum Bodyweight Exercise Repetitions Completed during a 20-min Exercise Bout	↑ Repetitions Completed (*d* = 0.33)	[40]
Jagim et al. 2016	12 resistance-trained male NCAA Division III football players	Randomized, Double-blind, placebo-controlled, crossover design.	^c^BCAAs (6000 mg) Citrulline Malate (6000 mg) Creatine (2000 mg) β –Alanine (2000 mg) Betaine (1500 mg) L-Tyrosine (1500 mg) Taurine (1000 mg) N-Acetyl L-Cysteine (600 mg) Caffeine (300 mg)	30 min prior to exercise	CMVJ, 5 × 5 bench press at 85% 5RM, 1 set of RTF at 85% 5RM, Anaerobic treadmill sprint test.	↑ BP RTF During Set 5 (*d* = 0.38) ↑ BP Total Volume Load (*d* = 0.19) ↑ Mean Sprint Power (*d* = 0.25) ↔ BS RTF ↔ BS Total Volume Load ↔ Lower Body Power	[5]
Magrini et al. 2016	31 recreationally-active males and females	Randomized, Double-blind, placebo-controlled, parallel design.	*Prop. Blend: (350 mg)* Caffeine (158 mg) L-Leucine B-Phenylethylamine HCl L-Valine L-Isoleucine N-Acetyl-L-Tyrosine Yohimbine 5-hydroxytryptophan	30 min prior to exercise	Pushup RTF	↔ Pushup RTF	[34]
Martinez et al. 2016	13 recreationally-trained males	Randomized, Double-blind, placebo-controlled, crossover design.	^b^ *Prop. Blend 1: (3500 mg)* β-alanine (2000 mg) L-Tyrosine *Prop. Blend 2: (2000 mg)* Creatine Arginine BCAAs *Prop. Blend 3: (1750 mg)* Choline Bitartrate Glucuronolactone Caffeine	20 min prior to exercise	Medicine Ball Put, VJ, BP 1RM, WAnT	↑ WAnT Peak Power (*d* = 0.30) ↑ WAnT Mean Power (*d* = 0.24) ↔ MBP Performance ↔ VJ ↔ BP 1RM	[41]
Collins et al. 2017	25 recreationally-active males and females	Randomized, Double-blind, placebo-controlled, crossover design.	β-alanine (2100 mg) Arginine nitrate (1300 mg) Folic acid (325 mcg) Caffeine (200 mg) Niacin (65 mg) Vitamin B12 (45 mcg)	30 min prior to exercise.	BP and LP 1RM, 2 × 10 BP and LP at 70% 1RM, BP and LP RTF at 70% 1RM, 4 km cycle TT	↑ BP RTF (*d* = 0.57) ↑ LP RTF (*d* = 0.55) ↑ BP Volume (*d* = 0.34) ↑ Total Lifting Volume (*d* = 0.50) ↑ Retention of LP Force Production (NA) ↔ Retention of BP Force Production ↔ TT Performance ↔ LP Volume	[23]
Jung et al. 2017	25 recreationally-active males and females	Randomized, Double-blind, placebo-controlled, crossover design.	*Supplement 1:* β-alanine (3000 mg) Creatine (2000 mg) L-arginine (2000 mg) N-Acetyl-L-tyrosine (300 mg) Caffeine (284 mg) *Mucana pruitiens* extract (15 mg) *Supplement 2:* Supplement 1 + *Citrus aurantium* extract (20 mg)	60 min prior to exercise.	2 × 10 BP and LP at 70% 1RM, BP and LP RTF at 70% 1RM, WAnT, Stroop Test	Supplement 2: ↑ Resting Metabolic Rate (*d* = 0.71) ↑ Respiratory Exchange Ratio (*d* = 1.14–1.66) ↑ Stroop Test Performance (*d* = 0.36–0.69) ↔ BP RTF ↔ LP RTF ↔ WAnT Power	[39]
Tinsley et al. 2017	21 resistance-trained males and females	Randomized, Double-blind, placebo-controlled, crossover design.	*Supplement 1:* Creatine (3000 mg) Betaine (2500 mg) L-Carnitine L-Tartrate (1000 mg) *Rhodiola rosea* extract (180 mg) Huperzine (200 mcg) ^c^ *Supplement 2:* BCAAs (6000 mg) Citrulline Malate (6000 mg) Creatine (2000 mg) β –Alanine (2000 mg) Betaine (1500 mg) L-Tyrosine (1500 mg) Taurine (1000 mg) N-Acetyl L-Cysteine (600 mg) Caffeine (300 mg)	30 min prior to exercise.	Isokinetic squat MVC, 4 × 6 isokinetic squats	↔ Squat MVC	[32]
Bergstrom et al. 2018	12 resistance-trained males	Randomized, Double-blind, placebo-controlled, crossover design.	Citrulline Malate (6000 mg) L-Leucine (4000 mg) D-Aspartic Acid (3000 mg) Creatine (2000 mg) β-alanine (1600 mg) L-Tyrosine (1200 mg) Caffeine (350 mg)	30 min prior to exercise.	8 resistance exercises, 4 sets of RTF at 75% 1RM followed by: Isokinetic MVC, VJ, Bench Throw, Cycle Critical Power and Anaerobic Capacity Tests	↑ Total Exercise Volume (*d* = 0.42) ↑ Lower Body Exercise Volume (*d* = 0.57) ↔ Upper-body Exercise Volume ↔ Isokinetic MVC ↔ Bench Throw ↔ Critical Power	[33]
Cameron et al. 2018	15 recreationally- active females	Randomized, Double-blind, placebo-controlled, crossover design.	*Prop Blend: (5850 mg)* β-alanine Choline Bitartrate L-Tyrosine L-Glycine Taurine L-Carnitine Betaine Hawthorn Berry Agmatine Sulfate Caffeine	60 min prior to exercise.	BP and BS RTF at 85% 5RM, CMVJ, Treadmill Sprint Test	↑ Bench Press RTF (RE = 107.66) ↑ Total Work during Sprint (*d* = 0.14) ↑ Resting Metabolic Rate (*d* = 1.64) ↔ Back Squat RTF ↔ CMVJ	[4]
Erickson et al. 2018	12 aerobically-trained females	Randomized, Double-blind, placebo-controlled, crossover design.	^d^ *Prop. Blend 1: (1000 mg)* L-Carnitine Green Coffee Bean Extract Cayenne Fruit Extract Forskolin *Prop. Blend 2: (371 mg)* N-Acetyl-L-Tyrosine Caffeine (150 mg) *Mucana pruitiens* extract (15 mg) β-alanine (1600 mg) Arginine (1000 mg)	30 min prior to exercise.	30 min constant velocity treadmill running at 90% VT	↔ Fat oxidation	[47]
Hahn et al. 2018	14 recreationally-active males	Randomized, Double-blind, placebo-controlled, crossover design.	L-Tyrosine (500 mg) Taurine (200 mg) Caffeine (120 mg) L-Carnitine (10 mg) Vitamin B6 (15 mg) Vitamin B12 (45 mcg)	20 min prior to exercise.	CMVJ, Treadmill Sprint Test	↔ Average Power ↔ Peak Power	[42]
Lane et al. 2018	23 recreationally-active males	Randomized, Double-blind, placebo-controlled, crossover design.	L-Citrulline DL-Malate (3000 mg) β-alanine (2000 mg) BCAAs (1500 mg) Creatine (1500 mg) Caffeine (300 mg) Vitamin B6 (20 mg) Vitamin B12 (500 mcg)	20 min prior to exercise.	CMVJ, Repeated Cycle Sprint Test, 10 × 3 bench press at 80% 1RM	↑ Bench Press Peak Velocity (*d* = 0.83) ↑ Bench Press Mean Velocity (*d* = 0.86) ↔ Cycle Sprint Power ↔ CMVJ	[6]
Musgjerd et al. 2018	20 aerobically-trained males and females	Randomized, Double-blind, placebo-controlled, crossover design.	^d^ *Prop. Blend 1: (1000 mg)* L-Carnitine Green Coffee Bean Extract Cayenne Fruit Extract Forskolin *Prop. Blend 2: (371 mg)* N-Acetyl-L-Tyrosine Caffeine (150 mg) *Mucana pruitiens* extract (15 mg) β-alanine (1600 mg) Arginine (1000 mg)	30 min prior to exercise	Simulated 5 km running race	↔ Running performance	[45]

↑ = MIPS was significantly greater (*p* < 0.05) than control; ↓ = MIPS was significantly less (*p* < 0.05) than control; ↔ = No significant difference (*p* > 0.05) was observed between MIPS and control; *1RM* 1 repetition maximum, *BP* Bench press, *BS* Back Squat, *CMVJ* Countermovement vertical jump, *d* Effect Size, *km* kilometer, *LP* Leg press, *MBP* Medicine ball put, *mg* milligram, *mcg* microgram, *MVC* Maximal voluntary contraction, *RE* Relative Effects, *RTF* repetitions to fatigue, *VO*_*2max*_ maximal oxygen consumption, *VJ* Vertical jump, *VT* Ventilatory threshold, *TT* Time trial, *TTE* time to exhaustion, *WAnT* Wingate anaerobic cycle test

^a^Studies used an identical MIPS containing BCAAs, β-alanine, caffeine, and creatine

^b^Studies used similar formulations of a MIPS containing BCAAs, β-alanine, caffeine, creatine, and citrulline malate

^c^Studies used an identical MIPS containing BCAAs, β-alanine, caffeine, creatine, citrulline malate, betaine, and tyrosine

^d^Studies used an identical MIPS containing β-alanine, caffeine, L-arginine, L-tyrosine and *Mucana pruitiens* extract (15% L-Dopa)

### Force production

Acute MIPS ingestion appears to have little effect on maximal force production (i.e, strength), though results of several recent investigations suggest that pre-exercise consumption of a caffeine-containing MIPS may help to mitigate fatigue-related decrements in force production experienced over a series of repetitions. For example, Tinsley et al. [32] noted that consumption of either caffeine-free or caffeine-containing pre-workout supplements did not improve maximal concentric or eccentric force production during squatting exercise in both males and females. Collins and colleagues [23] employed a crossover design to examine the effect of a caffeine-containing ready-to-drink pre-workout beverage on bench press and leg press one-repetition maximum (1RM) and muscular endurance testing. During the acute testing phase, male and female study participants performed bench press and leg press 1RM and muscular endurance testing (3 sets of 10 repetitions, last set to fatigue) to induce fatigue and then consumed either MIPS or placebo. The researchers noted that MIPS consumption prevented a decline in post-supplementation leg press 1RM following the pre-fatiguing bout of exercise and tended to mitigate decrements in bench press 1RM, though this effect was not statistically significant. Likewise, Bergstrom et al. [33] reported no differences in maximal isokinetic force production between males who consumed MIPS or placebo when measured after a training session containing high-volume resistance exercise to fatigue. However, the lack of between-group differences is notable, as the MIPS group had performed 9–14% more total volume (*d* = 0.42, *p* = 0.004, small) during the session compared to the group who had consumed placebo. Therefore, while more research is warranted in this area, it appears that consumption of MIPS may allow for greater retention of force production during and after exercise.

### Muscular endurance/Total volume completed

Many of the studies investigating the effect of MIPS on muscular endurance have reported that subjects who consumed MIPS completed more resistance exercise repetitions to fatigue relative to those who consumed placebo, though disparities between study designs, supplement contents, and dosing protocols make direct comparisons challenging. Hoffman and colleagues [16] noted that males who consumed a MIPS after a 7-day creatine monohydrate loading period performed significantly more back squat repetitions to fatigue compared to placebo after prior completion of 5 sets of 10 repetitions at 75% 1RM (NA). Conversely, Magrini et al. [34] failed to show between-group differences in push-up repetitions to fatigue following consumption of caffeine-containing MIPS or placebo. These null results were mirrored by Bloomer and colleagues [35], who evaluated the performance effects of three commercially-available, caffeine-containing MIPS on repetitions completed to fatigue using a bench press exercise machine. The researchers found no performance benefits of MIPS compared to placebo and noted that the dosage of caffeine contained in the proprietary blend of each MIPS was likely insufficient to confer ergogenic effects. Finally, Hoffman and colleagues [36] found no effect of acute MIPS supplementation on maximum pushups or sit-ups completed within a minute.

Interestingly, several investigations have reported that MIPS consumption improved either upper or lower-body muscular endurance, but not both within the same participants. Cameron and associates [4] found that female subjects who consumed a caffeine-containing MIPS completed significantly more bench press repetitions to fatigue compared to placebo (RE = 107.66, *p* = 0.037), though no between-group differences were observed for back squat repetitions to fatigue. Likewise, Jagim et al. [5] noted that male participants performed significantly more bench press (*d* = 0.38, *p* = 0.027, small) but not back squat repetitions to fatigue compared to placebo after consuming MIPS. Conversely, Spradley and colleagues [37] reported that a pre-workout supplement significantly improved leg press but not bench press repetitions to fatigue at 75% 1RM (*d* = 0.42–0.72, *p* < 0.024, small - moderate). However, Collins and associates [23], observed that acute MIPS consumption significantly increased both bench press (*d* = 0.57, *p* < 0.05, moderate) and back squat (*d* = 0.55, *p* < 0.05, moderate) repetitions to fatigue at 70% 1RM compared to pre-supplementation values. Such an increase is particularly notable, since the participants were able to improve upon pre-testing values even after fatiguing exercise. Thus, while mixed results have been reported, the consensus is that consumption of MIPS may significantly augment muscular endurance performance as measured by repetitions to fatigue.

Similarly, acute MIPS usage appears to augment the total exercise volume completed by participants in a variety of open-ended exercise modalities where maximal performance is encouraged. Jagim et al. [5] reported that acute MIPS ingestion significantly increased total volume load completed by participants (defined as total repetitions completed x load) during a bench press set to fatigue at 85% of 5RM (*d* = 0.19, *p* = 0.032, very weak), though no such effect was found for back squat using an identical protocol. Likewise, Collins and colleagues [23] found that acute consumption of a ready-to-drink MIPS prior to open-ended bench press and leg press tests increased both bench press volume (*d* = 0.34, *p* < 0.05, small) and total lifting volume (*d* = 0.50, *p* < 0.05, moderate) relative to fat-free mass completed by the participants. These results were supported by Gonzalez et al. [38], who noted that total lifting volume completed during four sets of barbell back squat or bench press at 80% 1RM was significantly augmented (*d* = 0.30, *p* = 0.022, small) following consumption of a MIPS 10 minutes prior to exercise. Bergstrom and colleagues [33] likewise demonstrated that pre-exercise consumption of a MIPS resulted in significantly greater total (*d* = 0.42, *p* = 0.004, small) and lower-body volume load (*d* = 0.57, *p* = 0.010, moderate) completed during a session consisting of four lower-body and four upper-body multi-joint barbell exercises, with four sets to fatigue at 70% 1RM being performed for each exercise. However, the researchers noted that upper-body volume was unchanged relative to placebo.

Conversely, Bloomer et al. [35] noted that both total and mean volume load performed during 10 sets of repetitions to fatigue at 50% 1RM using a bench press machine were unaffected by MIPS consumption. Similarly, Jung and associates [39] reported that the pre-exercise consumption of a caffeine-containing MIPS both with and without 20 mg synephrine had no effect on total lifting volume completed during bench press and leg press repetition tests to fatigue at 70% 1RM. However, bench press lifting volume tended to be greater during the third set of the MIPS + synephrine condition. The effect of MIPS consumption on total volume completed during other open-ended exercise modalities has received less attention, though several recent studies have reported promising results. For example, Cameron et al. [4] found that a caffeine-containing MIPS consumed 1 hour prior to sprint running significantly increased the total distance covered during a 25 s maximal-effort sprint test on a force-treadmill (*d* = 0.14, *p* = 0.039, very weak). Likewise, Jacobs et al. [40] observed that MIPS supplementation resulted in significantly greater volume of bodyweight exercise completed by male and female participants during a 20-min timed high-intensity bodyweight workout bout (*d* = 0.33, *p* < 0.05, small).

### Power production

Conflicting results have been reported regarding the effect of MIPS on upper-body and lower-body power production, though preliminary evidence has suggested that consumption of MIPS may help to retain upper-body power production following exercise. Bergstrom and colleagues [33] found that bench press throw barbell velocity assessed after a fatiguing bout of resistance exercise was similar to baseline measures when subjects consumed MIPS pre-exercise, but was significantly decreased (*d* = − 0.58, *p* < 0.05, moderate) following consumption of placebo. Other investigations have assessed the effect of MIPS supplementation on barbell velocity during resistance exercise, with mixed results. Gonzalez et al. [38] reported that consumption of MIPS resulted in increased average peak (*d* = 0.53, *p* < 0.001, moderate) and mean power performance (*d* = 0.29, *p* < 0.001, small) assessed via a linear position transducer during four sets of multi-joint exercise to fatigue. Lane and Byrd [6] investigated the effects of acute MIPS consumption in recreationally-trained males and likewise found that acute consumption of a MIPS as well as a caffeine-matched placebo resulted in increased peak velocity during bench press exercise (*d* = 0.83–0.86, *p* < 0.046, large) compared to placebo alone. Conversely, Jagim and associates [5] found no treatment effect of MIPS on either peak and average power measured with a linear position transducer during six sets of Smith machine back squat and bench press exercise. These results are in accordance with the findings of Bloomer and colleagues [35], who noted that the consumption of three types of MIPS had no effect on upper body power as measured by bench press throws at 30% 1RM. Martinez et al. [41] likewise failed to show a treatment effect of acute MIPS consumption on performance during a medicine ball put test, which requires participants to throw a medicine ball as far as possible.

Pre-exercise MIPS consumption appears to improve anaerobic sprinting performance in certain cases, though results are inconclusive. Jagim and associates [5] observed significant increases in mean power production (*d* = 0.25, *p* < 0.034, small) following MIPS ingestion during a 25-s maximal effort sprint test on a non-motorized treadmill set to a resistance equal to 18% bodyweight. However, later investigations by the same research group using similar treadmill testing protocols found no between-group differences in either peak or mean power production during the sprinting tests in both male [42] and female [4] subjects following consumption of two different MIPS products. Spradley et al. [37] likewise failed to show an effect of MIPS consumption on anaerobic running capacity as assessed by intermittent critical velocity testing. Conflicting results have likewise been reported regarding the effect of MIPS on anaerobic cycling performance. Martinez and associates [41] reported that peak and mean power measured during a Wingate anaerobic test (30 s all-out cycling with a resistance equal to 7.5% bodyweight) were significantly increased (*d* = 0.24–0.30, *p* < 0.006, small) following consumption of MIPS. Conversely, Jung et al. [39] as well as Hoffman et al. [36, 43] found no effect of acute MIPS consumption on Wingate anaerobic cycling performance. Lane and Byrd [6] also failed to show an ergogenic effect of MIPS or a caffeine-matched placebo on repeated 5 s Wingate cycling performance.

Similarly, it appears that MIPS ingestion has little effect on jumping performance, as three recent studies conducted by the same research group found no treatment effect of MIPS on countermovement vertical jump height or power production in male [5, 42] and female [4] subjects following acute ingestion. These results were also supported by Lane and Byrd [6], who found no effect of acute MIPS supplementation on vertical jump performance.

### Endurance exercise performance

Limited evidence exists regarding the effect of acute MIPS ingestion on endurance exercise performance. To date, only one study has investigated the effect of acute MIPS supplementation on time to exhaustion during endurance exercise, with promising results. Walsh and colleagues [44] utilized a randomized, double-blind, crossover design to assess the effect of a caffeine-containing MIPS on time to exhaustion during treadmill exercise performed at a customized velocity and grade that elicited 70% of each subject’s VO_2_max. The researchers found that time to exhaustion was significantly greater following MIPS consumption compared to placebo, as subjects were able to run 12.5% longer (NA) after consuming the supplement 10 min before exercise. However, a recent study by Collins et al. [23] found that acute MIPS consumption had little effect on cycling time-trial performance in resistance-trained males and females. Following a one-minute warmup with gradually increasing load, participants performed a 4 km time trial as quickly as possible using a standardized resistance of 4 J/kg/rev. No differences in time, wattage, or relative wattage were observed between conditions. However, because participants completed the time trial faster after consumption of MIPS compared to placebo, the researchers noted that the MIPS did not result in an ergolytic effect on performance. Similarly, Musgjerd and colleagues [45] noted that acute MIPS supplementation had no effect on running performance during a simulated 5 km running race. Clearly, more information is needed before results can be generalized further.

### Subjective responses

Acute MIPS ingestion appears to improve self-reported subjective measures of focus, fatigue, alertness and self-reported energy levels, though conflicting results have been reported. Hoffman and colleagues [43] noted that pre-exercise consumption of a caffeine-containing supplement resulted in significantly increased subjective feelings of energy (*d* = 0.8, *p* < 0.05, large) and focus (*d* = 0.82, *p* < 0.05, large) as well as a trend towards increased alertness in male strength and power athletes during testing sessions that included repeated Wingate anaerobic cycle tests and reaction time testing. A 2010 study by the same research group [36] reported that recreationally-active subjects who consumed a caffeine-containing supplement prior to exhaustive exercise were better able to maintain focus and alertness (NA) compared to those who consumed placebo, though fatigue was elevated after exercise (NA) in the MIPS group but not the placebo group. A later study by Jagim et al. [5] using similar Likert-based subjective assessments found that subjects who consumed a caffeine-containing MIPS reported significantly lower subjective fatigue (*d* = − 3.78, *p* = 0.01, strong) and increased alertness (*d* = 2.72, *p* < 0.05, strong) following high-volume resistance exercise to fatigue. In addition, subjects in the treatment group reported significantly greater feelings of focus following completion of maximal treadmill sprinting (*d* = 2.78, *p* = 0.01, strong). A similar study by the same research group [4] found that females who ingested a caffeine-containing MIPS reported significantly greater feelings of focus (NA) at 80 min after ingestion compared to placebo, though no effect was found on feelings of energy and fatigue. Likewise, Spradley and colleagues [37] found that self-reported energy was higher (*d* = 1.34, *p* < 0.05, strong) and fatigue significantly lower (*d* = − 1.46, *p* < 0.05, strong) in recreationally-trained males at the midpoint of the testing session, approximately 95 min after consumption of a MIPS. The researchers reported significant main effects of treatment for self-reported energy (*d* = 0.35, *p* < 0.05, small), focus (*d* = 0.71, *p* < 0.05, moderate), and alertness (*d* = 0.53, *p* < 0.05, moderate) across the entire testing session, which included resistance exercise and reaction time drills. Similar results were observed by Walsh et al. [44], who found that subjects who consumed MIPS reported significantly greater feelings of focus (*d* = 0.54, *p* = 0.031, moderate) and energy (*d* = 0.49, *p* = 0.016, small) and less fatigue (*d* = 0.60, *p* = 0.005, moderate) immediately before treadmill exercise as well as greater feelings of focus (*d* = 0.67, *p* = 0.026, moderate) and energy (*d* = 1.26, *p* = 0.004, strong) 10 minutes after the start of a treadmill time to exhaustion protocol at 70% VO_2_max. No between-group differences were found after completion of the exercise protocol. Jung and colleagues [39] also observed that participants who consumed MIPS with or without added synephrine reported they felt more vigorous and energetic (*d* = 0.52, *p* < 0.05, moderate) and were more optimistic about their future performance (*d* = 0.26–0.32, *p* < 0.05, small) compared to those who consumed placebo, though those who consumed MIPS with added synephrine reported significantly lower feelings of vigor and energy immediately after testing (*d* = − 0.55, *p* < 0.05, moderate) compared to participants who consumed placebo or MIPS alone.

Conversely, Hahn and colleagues [42] reported that self-reported feelings of fatigue following maximal-effort treadmill sprinting were significantly elevated after consumption of both MIPS (*d* = 1.16, *p* < 0.05, strong) and placebo (*d* = 2.86, *p* < 0.05, strong), though the magnitude of the change from baseline was significantly lower (*d* = − 1.10, *p* < 0.05, strong) in the MIPS condition compared to placebo. Additionally, Gonzalez et al. [38] found no effect of MIPS on ratings of energy, fatigue, or focus during a bout of multi-joint resistance exercise to fatigue; findings which were replicated by Tinsley et al. [32] who did not detect between-group differences in any self-reported subjective variables during a bout of isokinetic squat exercise. Finally, Musgjerd and colleagues [45] demonstrated that acute MIPS consumption had no effect on subjective measures of fatigue, alertness, energy, or focus either before or after a 5 km simulated running competition. In contrast to other investigations which examined self-reported mood state and fatigue, Bloomer and colleagues [35] instructed subjects to rate their perceived muscle “pump” before and after exercise. The researchers defined “pump” as a feeling of size, hardness, and swelling within the muscle. While the subjects reported increased levels of “pump” after exercise, no between-group differences were noted. There appears to be a general trend regarding a positive influence on subjective ratings of energy and fatigue following MIPS ingestion, which could play a role in the overall quality of a training session and improve exercise tolerability for certain individuals.

### Reaction time and cognitive processing

The results of several investigations suggest that acute MIPS consumption may have a beneficial effect on reaction time in recreationally-trained [36, 37] and resistance-trained [43] males. Hoffman and colleagues [43] investigated the acute effects of a ready-to-drink caffeine-containing MIPS on reaction time performance during three tests which were interspersed with 20-s Wingate Anaerobic cycle tests. The researchers found that MIPS resulted in significant improvements in the number of targets struck during the test (*d* = 0.53, *p* < 0.05, moderate) as well as percent of targets struck (*d* = 0.48, *p* < 0.05, small) relative to placebo. A similar investigation by the same group [36] found that recreationally-active participants who consumed MIPS were better able to maintain reaction time (NA) after a bout of exhaustive exercise compared to those who consumed placebo. These results were supported by Spradley et al. [37] in a later study, who found that acute MIPS consumption resulted in significantly improved reaction time performance during a 30 s test (*d* = − 0.33 - -0.46, *p* < 0.05, small) using combined visual and auditory stimuli. Furthermore, Jung and colleagues [39] found that acute consumption of a MIPS with added synephrine significantly improved performance during a Stroop test (*d* = 0.36–0.69, *p* < 0.037, small to moderate), a type of standardized cognitive function test that measures reaction time, selective attention, and cognitive processing. Thus, while more information is required in this area, initial results are promising.

### Hormonal response to resistance exercise

The effect of acute MIPS consumption on the short-term hormonal response to resistance exercise is unclear; as mixed results have been reported in the literature. Ratamess et al. [46] reported that MIPS consumed 20 min before 6 sets of 10 repetitions of back squat exercise at 75% 1RM had no effect on serum levels of total testosterone and growth hormone (GH) in resistance-trained males during 30 min of recovery. However, a later study by the same research group [16] using a similar design and population found that subjects who consumed the same MIPS after 1 week of creatine loading had significant improvements in post-exercise serum growth hormone (NA), total testosterone (NA), and insulin levels (NA) compared to those who consumed placebo after an identical creatine loading protocol. Clearly, more information is needed in this area.

### Energy expenditure and substrate utilization

Acute MIPS consumption may affect energy expenditure as well as substrate utilization at rest, though conflicting results have been reported. Similarly, limited evidence exists regarding the effect of MIPS on substrate utilization during prolonged aerobic exercise. Cameron and colleagues [4] reported that acute MIPS consumption resulted in higher resting energy expenditure (REE) (*d* = 1.64, *p* < 0.043, large) in recreationally-active females. Conversely, Jung et al. [39] found that a MIPS with added synephrine increased REE (*d* = 0.71, *p* < 0.05, large) and RER (*d* = 1.14–1.66, *p* < 0.05, large) in recreationally-active male and female participants relative to placebo during a supine resting metabolic rate test. However, Erickson and colleagues [47] found no effect of acute MIPS consumption on fat oxidation in females during 30 min of treadmill running at 90% ventilatory threshold. Clearly, more investigations in a wider variety of populations are required before definite conclusions can be determined regarding any metabolic-related benefits.

### Gender

Limited evidence exists regarding a gender effect of acute MIPS supplementation, as none of the eight acute MIPS studies which examined the effects of MIPS on a mixed gender cohort [23, 32, 34, 36, 39, 40, 44, 45] reported any form of menstrual control, and only two of these investigations [23, 32] used gender as a covariate in their analyses. Collins and colleagues [23] used gender and relative caffeine intake as covariates in their analysis to control for the influence of the menstrual cycle and birth control, though they noted that no time x treatment x gender interaction effects were found for any outcome variable. Similarly, Tinsley et al. [32] did not detect any time x treatment x gender interaction effects, suggesting that any gender effect of MIPS supplementation is minimal. In addition, two investigations [4, 47] have examined the effects of acute MIPS supplementation in female participants only. While both did not report any form of menstrual control, the results of these studies are not particularly divergent from those conducted in males. Cameron et al. [4] found MIPS increased upper-body muscular endurance but had no effect on lower-body muscular endurance or power production, while Erickson et al. [47] reported that acute MIPS supplementation had no appreciable effect on rates of fat oxidation. However, any conclusions regarding a gender effect of acute MIPS supplementation are likely premature until more robust studies that utilize menstrual control are conducted.

## Chronic effects of MIPS on performance

Many investigations have employed short-term (10 days or less) as well as long-term (greater than 10 days) supplement interventions both with and without a concurrent training intervention. Studies including a training intervention are termed ‘training studies,’ while those that employed a single testing bout of exercise are termed ‘supplement loading studies.’ Additionally, some studies used protocols which incorporated MIPS ingestion on the day of post-testing. These studies and their relevant supplementation protocols are outlined in Tables 2 and 3.

**Table 2:** Performance outcomes and training adaptations in short-term (7–10 days) MIPS studies

Study	Subjects	Design	Supplement Ingredients (Sorted by order listed on Supplement Facts label)	Timing	Training Protocol	Performance Testing Protocol	Results (*d*)	Ref #
Kraemer et al. 2007	9 resistance trained males	Double-blind, placebo controlled, cross-over, 7-day supplementation study	L-Arginine (2000 mg) D-Ribose (1500 mg) L-Carnitine (400 mg) L-Citrulline (200 mg) Betaine (100 mg) Caffeine (70 mg)	30 min prior to exercise or at a similar time on off days	NA	CMVJ, six sets of BS RTF at 80% 1RM, isometric squat force^1^	↑ Power (NA) ↑ RTF for 5 of 6 sets (*d* = 0.41–1.08)	[49]
Outlaw et al. 2014	20 resistance trained males	Double-blind, placebo controlled, training study	*Prop. Blend: (8400 mg)* Creatine β-alanine BCAAs (4800 mg) Caffeine (275 mg)	30 min prior to exercise or during the morning hours on off days	8 days of supplementation with four split body training sessions	DEXA, VJ, BP & LP 1RM, WAnT^1^	↔ Power ↔ Body Composition ↔ BP 1RM ↔ LP 1RM	[48]
Collins et al. 2017	25 resistance trained males and females	Double-blind, placebo controlled, 7-day supplementation study	β-alanine (2100 mg) Arginine nitrate (1300 mg) Folic acid (325 mcg) Caffeine (200 mg) Niacin (65 mg) Vitamin B12 (45 mcg)	Unreported time point during supplementation phase	NA	BP and LP 1RM, 2 × 10 BP and LP at 70% 1RM, BP and LP RTF at 70% 1RM, 4 km cycle TT^1^	↔ Retention of force production ↔ Muscular endurance ↔ Lifting volume ↔ Power	[23]
Martin et al. 2017	30 resistance trained males	Double-blind, placebo controlled, supplementation study	Creatine (2000 mg) Β-alanine (1600 mg) L-Citrulline (1000 mg) Caffeine (300 mg) L-Norvaline (200 mg) L-Theanine (100 mg) Whole coffee fruit extract (100 mg) Theacrine (50 mg)	Unreported time point during supplementation phase	NA	Leg extension RTF at 30% or 80% 1RM^1^	↔ RTF ↔ Volume load	[50]

↑ = MIPS was significantly greater (*p* < 0.05) than control; ↓ = MIPS was significantly less (*p* < 0.05) than control; ↔ = No significant difference (*p* > 0.05) was observed between MIPS and control; *1RM* 1 repetition maximum, *%BF* body fat percentage, *BP* bench press, *BS* back squat, *CMVJ* counter-movement vertical jump, *d* Effect Size, *DEXA* dual x-ray energy absorptiometry, *FFM* fat-free mass, *FM* fat mass, *LP* Leg press, *mg* milligram, *mcg* microgram, *POMS* Profile of Mood States, *RE* Relative Effects, *RTF* repetitions to fatigue, *WAnT* Wingate anaerobic cycle test, *VJ* vertical jump

^1^Testing performed with acute MIPS supplementation

**Table 3:** Performance outcomes and training adaptations in long-term (> 10 day) MIPS studies

Study	Subjects	Design	Supplement Ingredients (Sorted by order listed on Supplement Facts label)	Timing	Training Protocol	Performance Testing Protocol	Results (*d* or RE)	Ref #
Stout et al. 2008	38 sedentary males and females	Double-blind, placebo controlled, resistance and endurance training study	*Prop. Blend: (1810 mg)* Taurine Guarana extract Green tea leaf extract Caffeine Glucuronolactone Ginger	15 min prior to exercise or ad libitum on off days	10 weeks of resistance and endurance training based on ACSM guidelines	Body composition, VO_2peak_, TTE^3^	↓ FM (NA) ↑ VO_2peak_ (NA) ↑ TTE (NA)	[62]
Hoffman et al. 2010	19 recreationally-active males and females	Randomized, Double-blind, placebo-controlled supplementation study.	Acetyl-L-carnitine (500 mg) L-tyrosine (500 mg) Α-glycerophosphocholine (150 mg) Choline bitartrate (125 mg) Caffeine (60 mg) Phosphatidylserine (50 mg)	10 min prior to testing. Timing not specified on off days.	NA	Choice Reaction Time Test before and after the following: WAnT, Maximum Pushup Repetitions Completed in one Minute, Maximum Situp Repetitions Completed in one Minute^1^	↔ Reaction Time ↔ Peak Power ↔ Mean Power ↔ Pushups Completed ↔ Sit-ups Completed	[36]
Shelmadine et al. 2009	18 non-resistance trained males	Double-blind, placebo controlled, resistance training study	^a^ *Prop. Blend 1: (5350 mg)* L-Leucine L-nor-Valine L-Valine L-Isoleucine *Prop. Blend 2: (10,952 mg)* Methyl Hydroxy Chalcone Polymer Creatine Arginine Glutamine Ariginine Citrulline Malate *Prop. Blend 3: (229 mg)* L-Tyrosine Caffeine	30 min prior to exercise or immediately upon waking on off days^3^	28-days of resistance training (4 sessions/week; upper/lower body split)	DEXA, muscle biopsies, BP & LP 1RM^3^	↑ FFM (*d =* 0.24) ↑ Relative BP 1RM (*d =* 0.23) ↔ Relative LP 1RM	[53]
Schmitz et al. 2010	20 resistance trained males	Double-blind resistance training study using comparator control	^c^ *Prop. Blend 1:* Whey protein (7 g) L-Leucine (4 g) L-Isoleucine L-Valine *Prop. Blend 2:* Creatine (4 g) *Prop. Blend 3:* Taurine L-alanyl-L-glutamine Magnesium glycyl glutamine *Comparator matched for carbohydrate, whey, and creatine	Begin 10–15 min prior to exercise and complete before end of session. No supplement on off days.	9 weeks of periodized resistance training (4 sessions/week; upper/lower body split)	DEXA, BP 1RM, 3 sets of RTF at pretesting body weight^3^	↑ BP 1RM (RE = 106.5) ↑ RTF (RE = 131.8) ↓ FM (RE = 87.1) ↑ FFM (RE = 102.1)	[51]
Smith et al. 2010	24 recreationally trained males	Single-blind, placebo controlled, HIIT training study	*Prop. Blend: (2100 mg)* *Cordyceps sinensis* Arginine Creatine Citrulline Taurine Leucine Valine Isoleucine Caffeine Whey protein	30 min prior to exercise/testing. Nothing on off days.	3 weeks of HIIT interval training (3 sessions/week)	BodPod, VO_2max_ followed by four sprints to exhaustion^1^	↑ Training volume (NA) ↔ FFM ↔ VO_2max,_ critical velocity	[63]
Spillane et al. 2011	19 non-resistance trained males	Double-blind, placebo controlled, resistance training study	^a^ *Prop. Blend 1: (5350 mg)* L-Leucine L-nor-Valine L-Valine L-Isoleucine *Prop. Blend 2: (10,952 mg)* Methyl Hydroxy Chalcone Polymer Creatine Arginine Glutamine Ariginine Citrulline Malate *Prop. Blend 3: (229 mg)* L-Tyrosine Caffeine *Subjects also consumed a post-workout supplement	30 min prior (pre) and within 30 min of end of exercise (post). Immediately upon waking on off days (post).	28 days of resistance training (4 sessions/week)	DEXA, BP & LP 1RM, muscle biopsies^3^	↓ FM (*d* = − 0.08) ↑ FFM (*d* = 0.17) ↑ Myofibrillar protein mass (*d* = 0.32) ↑ Upper (*d* = 0.57), lower-body (*d* = 0.62) relative strength	[54]
Ormsbee et al. 2012	24 resistance trained males	Double-blind, placebo controlled, resistance training study	^b^ *Prop. Blend 1: (9660 mg)* Casein Protein Whey Protein BCAAs L-arginine *Prop. Blend 2: (4890 mg)* Creatine Citrulline Malate Arginine *Prop. Blend 3: (3170 mg)* β-alanine Creatine *Prop. Blend 4: (376 mg)* Caffeine *Subjects also consumed a post-workout supplement	15 min prior to exercise (pre) and immediately after end of exercise (post). Ad libitum on off days (post).	6 weeks of split resistance training (3 sessions/week)	DEXA, BP & LP 1RM, Isokinetic strength testing, WAnT^2^	↑ FFM (*d* = 0.18) ↔ Peak power ↔ BP 1RM ↔ LP 1RM	[55]
Willems et al. 2012	16 resistance trained males	Double-blind, placebo controlled, training study	Whey protein (30 g) Creatine (5.1 g) Glutamine (5.1 g) HMB (1.5 g)	One dose in the morning and another within 15 min of exercise end. Similar time on off days.	2, 6-week blocks of progressive resistance training (4 sessions/week)	BP, LP, & lateral pull 1-RM and RTF at 80% 1RM, peak isokinetic torque measured via a dynamometer, MVC^3^	↑ BP RTF* (*d* = 1.2) ↑ Lateral pull RTF* (*d* = 1.3) ↔ 1RM* ↔ MVC, peak concentric strength*	[58]
Lowery et al. 2013	20 resistance trained males	Double-blind, placebo controlled, resistance training study	*Prop. Blend 1: (2800 mg)* Creatine β-alanine *Prop. Blend 2: (1600 mg)* L-Taurine BCAAs Alanyl-glutamine *Prop. Blend 3: (620 mg)* Glucuronolactone Caffeine *Prop. Blend 4: (500 mg)* Natural nitrates Quercetin (67 mg)	30 min prior and 3 servings throughout the day on off days.	8 weeks of split focused resistance training (3sessions/week)	DEXA, BP & LP 1RM, ultrasonography^3^	↑ FFM (RE = 104.1) ↑ Quadriceps muscle thickness (RE = 108.0) ↑ BP 1RM (NA) ↔ LP 1RM	[52]
Ormsbee et al. 2013	24 resistance trained males	Double-blind, Placebo-controlled, resistance training study	^b^ *Prop. Blend 1: (9660 mg)* Casein Protein Whey Protein BCAAs L-arginine *Prop. Blend 2: (4890 mg)* Creatine Citrulline Malate Arginine *Prop. Blend 3: (3170 mg)* β-alanine Creatine *Prop. Blend 4: (376 mg)* Caffeine *Subjects also consumed a post-workout supplement	15 min prior (pre) and immediately after exercise (post). Ad libitum on off days (post).	6 weeks of periodized resistance training (3 sessions/week)	DEXA, POMS^3^	↑ FFM (*d* = 0.22) ↔ FM ↔ POMS	[65]
Kedia et al. 2014	43 resistance trained males and females	Double-blind resistance training study using comparator control	*Prop. Blend 1: (4580 mg)* Creatine Betaine Citrulline *Dendrobium* extract Caffeine *Comparator product matched for energy and caffeine content	30 min prior to exercise and with breakfast on off days.	6 weeks of periodized resistance training (4 sessions/week; upper/lower body split)	DEXA, BP 1RM, VAS of energy & concentration^3^	↑ Energy & concentration (NA) ↔ FFM ↔ FM ↔ BP 1RM	[3]
Kendall et al. 2014	17 recreationally trained males	Double-blind, placebo controlled 4-week supplementation study	BCAAs (6 g) Creatine (5 g) β-alanine (4 g) Citrulline (1.5 g) Caffeine (300 mg)	20 min prior to exercise and ad libitum on off days.	NA	Skinfolds (Brozek equation), VO_2max_, BP & LP 1-RM, RTF at 75% 1RM^3^	↑ VT (% of VO_2max_) (*d* = 1.21) ↑ LP 1RM (*d* = 0.45) ↓ Relative VO_2max_ (*d* = − 0.52)	[57]
Spillane et al. 2014	24 resistance trained males	Double-blind, resistance training study using comparator control	^c^ *Prop. Blend 1:* Whey protein (7 g) L-Leucine (4 g) L-Isoleucine L-Valine *Prop. Blend 2:* Creatine (4 g) *Prop. Blend 3:* Taurine L-alanyl-L-glutamine Magnesium glycyl glutamine *Comparator matched for maltodextrose, whey protein, and creatine monohydrate	½ dose consumed 15 min prior to exercise, other ½ dose consumed at start of exercise. Nothing on off days.	6 weeks of periodized resistance training (4 sessions/week; upper/lower split)	DEXA, BP, LP, & knee extension 1RM, BP & LP RTF at 75% 1-RM, muscle biopsies^3^	↔ Body Composition ↔ 1RM ↔ RTF	[61]
Kreipke et al. 2015	27 resistance trained males	Double-blind, placebo controlled, resistance training study	BCAA (2500 mg) β-alanine (1020 mg) Long jack root (250 mg)	20 min prior and 2 h after exercise. With breakfast and lunch on off days.	4 weeks of progressive resistance training (4 sessions/week)	DEXA, squat, BP, deadlight 1RM, POMS^3^	↑ BP 1RM (*d* = 0.05) ↑ Total weight lifted (*d* = 0.15) ↑ Relative deadlift strength (FFM: *d* = 0.11; Total Mass: *d* = 0.08)	[59]
Ormsbee et al. 2015	20 trained male runners	Double-blind, placebo controlled, 4-week supplementation study	^b^ *Prop. Blend 1: (9660 mg)* Casein Protein Whey Protein BCAAs L-arginine *Prop. Blend 2: (4890 mg)* Creatine Citrulline Malate Arginine *Prop. Blend 3: (3170 mg)* β -alanine Creatine *Prop. Blend 4: (376 mg)* Caffeine	30 min prior to exercise or immediately upon waking on off days.	NA	Skinfolds, perceived soreness VAS, ROM, vertical jump, isokinetic and isometric strength using isokinetic dynamometer, VO_2max_, before and after a single bout of downhill running^1^	↔ Isometric and isokinetic strength ↔ Flexibility ↔ Power ↔ Perceived soreness	[56]
Köhne et al. 2016	8 trained female runners	Double-blind, placebo controlled, 28-day supplementation study	^b^ *Prop. Blend 1: (9660 mg)* Casein Protein Whey Protein BCAAs L-arginine *Prop. Blend 2: (4890 mg)* Creatine Citrulline Malate Arginine *Prop. Blend 3: (3170 mg)* β-alanine Creatine *Prop. Blend 4: (376 mg)* Caffeine *Subjects also consumed a post-workout supplement	30 min prior to exercise or immediately upon waking on off days.	NA	VO_2max_ (before and after supplementation), muscle pain perception, muscle damage markers, squat jump power, hamstring flexibility, and limb circumferences (before and after a single bout of downhill running after 28 days of supplementation)^1^	↔ Hamstring flexibility ↔ Squat jump power ↔ Limb circumference	[64]
Jung et al. 2017	80 resistance trained males	Double-blind, placebo controlled, resistance training study	*Supplement 1:* β-alanine (3000 mg) Creatine (2000 mg) L-arginine (2000 mg) N-Acetyl-L-tyrosine (300 mg) Caffeine (284 mg) *Mucana pruitiens* extract (15 mg) *Supplement 2:* Supplement 1 + *Citrus aurantium* extract (20 mg)	15–30 min prior to exercise or with breakfast on off days.	8 weeks of resistance training (4 sessions/week; upper/lower body split)	Cognitive function, readiness to perform, BP & LP 1RM, Wingate anaerobic cycling test^3^	↔ 1RM leg & bench press ↔ Power ↔ Readiness to perform ↑ Stroop Test Performance (*d* = 0.12–0.16)	[60]
Zabriskie et al. 2017	19 recreationally active females	Double blind, placebo controlled, resistance training study	*Prop. Blend 1: (5700 mg)* β-alanine L-tyrosine L-glycine L-carnitine Beet root Hawthorn berry powder Caffeine	30 min prior to exercise or after breakfast on off days.	7 weeks of periodized resistance training	BodPod, RMR^2^	↔ FM ↔ FFM ↔ RMR	[66]

↑ = MIPS was significantly greater (*p* < 0.05) than control; ↓ = MIPS was significantly less (*p* < 0.05) than control; ↔ = No significant difference (*p* > 0.05) was observed between MIPS and control; *1RM* 1 repetition maximum, *%BF* body fat percentage, *BP* Bench press, *d* Effect Size, *DEXA* dual x-ray energy absorptiometry, *FFM* fat-free mass, *FM* fat mass, *RE* Relative Effects, *RTF* repetitions to fatigue, *LP* Leg press, *mg* milligram, *mcg* microgram, *POMS* Profile of Mood States, *ROM* range of motion, *TTE* time to exhaustion, *VO*_*2max*_ maximal oxygen consumption, *VT* Ventilatory Threshold, *WAnT* Wingate anaerobic cycle test

^a^Studies used an identical MIPS containing whey protein, caffeine, BCAAs, creatine, β-alanine, and L-arginine

^b^Studies used a later formulation of the same brand name MIPS as ^a^ containing whey protein, caffeine, BCAAs, creatine, β-alanine, and L-arginine

^c^Studies used an identical MIPS containing carbohydrate, whey, creatine, BCAAs and L-taurine

^1^Testing performed with acute MIPS supplementation

^2^Testing performed without acute MIPS supplementation

^3^Presence of MIPS during testing not explicitly reported

*analysis performed using Cohen’s *d* effect sizes

### Short-term supplementation (< 10 days)

#### Force production

At best, short-term MIPS consumption might preserve 1-RM performance after several bouts of fatiguing exercise but does not appear to favorably impact maximal strength. For example, Collins et al. [23] performed a 6-day supplement loading study. During baseline testing, participants completed 1RM and muscular endurance (3 sets of 10 repetitions, last set to fatigue) testing of the bench and leg press before and after an acute dose of MIPS or placebo. All participants were instructed to consume MIPS for an additional 5 days, and on the sixth day, participants performed identical post-testing. The authors claim that placebo consumption resulted in significantly lower leg press 1-RM after exhaustive exercise, while those who had consumed MIPS for 5 days retained greater force production and thus had significantly greater 1RM performance (*d* = 0.18, *p* = 0.3, very weak). However, the combination of the large reported *p*-value and small effect size suggest that there was no difference between placebo and MIPS. Similarly, Outlaw et al. [48] found no influence of MIPS on bench press or leg press 1RM relative to placebo following an 8 day supplementation protocol coupled with four resistance training bouts. Given the minimal amount of research, it is difficult to conclude that any marked benefit of short-term MIPS supplement loading on force production is present.

#### Muscular endurance and total volume completed

Several studies have evaluated muscular endurance following a short-term supplement loading protocol, with somewhat promising results. Collins et al. [23] claimed that short term supplementation (5 days) with a MIPS tended to significantly increase repetitions to failure on the leg press compared to placebo (*d* = 0.455, *p* = 0.116, moderate) and improve leg press lifting volume after exhaustive exercise (*d* = 0.24, *p* = 0.157, weak). Again, however, the large *p*-values suggest that these claims are not evidenced by the results of this investigation. Similarly, Kraemer et al. [49] found that 7 days of MIPS supplementation significantly improved Smith machine squat repetitions to fatigue during 6 sets at 80% 1RM (*d* = 0.41–1.08, *p* < 0.05, moderate - strong). However, Martin and colleagues [50] found no effect of short-term MIPS supplementation on total repetitions or volume load completed during four sets of bilateral leg extensions to fatigue at both 30% and 80% 1RM. Again, given the minimal volume of research in this area, no solid conclusions can be made at this point in time.

#### Power production

A paucity of evidence exists regarding the effect of short-term MIPS supplementation on power production. Additionally, broad differences in study design make comparisons between the conclusions of relevant studies difficult. Kraemer and associates [49] found that individuals who had consumed MIPS for 7 days exhibited higher vertical jump power output than individuals consuming placebo (NA). These results may suggest that brief supplement loading may enhance performance during a single exercise bout. However, these findings contrast with those of Outlaw et al. [48], who found no difference in peak anaerobic power production during a 30-s Wingate Anaerobic Cycle test between subjects who consumed MIPS or placebo for 8 days. Collins et al. [23] similarly found no difference in anaerobic power output during a 4 km time trial in participants who had consumed MIPS for 5 days compared to placebo consumers. The difference in testing paradigms may potentially explain some of the discrepancy in findings, as the vertical jump depends largely on the ATP/PCr energy system, while a Wingate test draws upon both anaerobic energy systems. Clearly, more research is needed to investigate the effect of short-term MIPS supplementation on power production.

#### Serum hormones and markers of muscle damage

Limited evidence exists regarding the effect of short-term MIPS consumption on hormones or markers of muscle damage caused by acute exercise. Kraemer et al. [49] found no difference in lactate, glucose or insulin between MIPS or placebo users after 7 days of supplementation. However, serum levels of creatine kinase were significantly lower midway through squat exercise (*d* = − 0.85, *p* < 0.05, strong), immediately after exercise(*d* = − 0.76, *p* < 0.05, moderate), and during 15 min of recovery (*d* = − 0.61, *p* < 0.05, moderate), while myoglobin was reduced immediately following exercise (*d* = − 0.82, *p* < 0.05, strong) in MIPS users relative to placebo, potentially suggesting that the supplement may have affected muscle damage. Kraemer and colleagues [49] also found that MIPS consumption resulted in greater increases in GH (NA) and free testosterone (NA) midway during six sets of squats performed to fatigue at 80% 1RM and during 15 min of recovery (NA). Likewise, IGF-1 was elevated in the treatment group immediately before exercise (i.e. about 30 min after acute supplement ingestion) (NA), though insulin was no different between MIPS and placebo.

#### Gender

The only study included in this review which studied a female population consuming a MIPS for a short period of time is the investigation by Collins and colleagues [23]. Roughly half of their total sample size was female and gender was used as a covariate in their analysis. Though expected differences in variables such as height, body composition, absolute upper and lower body strength, and relative upper body strength were noted, researchers did not find the MIPS to exert a different effect on females than males over time. However, more research utilizing hormonal control is needed to fully understand the effects of gender on MIPS.

### Long-term supplementation (> 10 days)

A significantly greater volume of research exists that has investigated the benefits of MIPS supplementation lasting more than 10 days, typically in conjunction with some form of an exercise program. However, it is vital to note that not all of these investigations are placebo-controlled, as several studies opted to provide a comparator product lacking a proprietary blend of ingredients withheld by the supplement manufacturers as a control condition. However, these studies do not circumvent the central issue of differing proprietary blends and formulations in the various MIPS that were investigated, making it difficult to compare between products or identify primary active ingredients.

#### Force production

A substantial body of evidence suggests that long-term consumption of MIPS positively influences force production. It appears that long-term MIPS ingestion paired with resistance training results in increased 1-RM bench press strength compared to training alone in both trained (RE = 104.1–106.5, *p* < 0.02) [51, 52] and untrained [53, 54] males (*d* = 0.23–0.63, *p* < 0.05, weak - moderate). It is worth noting that both of the studies that found improvements in untrained males examined the same pre-workout supplement (containing a proprietary blend of caffeine, whey, BCAAs, creatine, β-alanine, and L-arginine) with a placebo control. However, one of the studies also required participants to consume a post-workout protein supplement, the addition of which may potentiate training adaptations beyond MIPS alone [54]. Regardless, these results suggest that chronic MIPS ingestion either with or without post-exercise protein will potentiate training adaptations resulting from a resistance exercise program. However, another investigator found no benefit of an identical MIPS on strength outcomes in resistance-trained males [55] or aerobically-trained males [56]. Notably, the resistance trained males consuming this specific MIPS also consumed a post-workout protein supplement, which also likely influenced training adaptations.

Interestingly, MIPS ingestion may improve upper-body strength but not lower-body strength despite lower-body resistance training, as evidenced by the work of Shelmadine et al. [53] and Lowery et al. [52], who both noted increases in bench press 1-RM but not leg press 1-RM in both trained and untrained males. Kendall et al. [57] likewise noted increases in leg press but not bench press strength after 4 weeks of supplementation loading without a supervised training intervention. However, due to the lack of supervised resistance training and the high volume of aerobic exercise conducted by the participants, these results are less generalizable and should be interpreted with caution. Conversely, Willems and associates [58] reported that MIPS supplementation resulted in no significant increases in upper or lower-body strength compared to placebo, though effect size analysis suggested that strength may have been increased in muscle groups and movements that were targeted during the resistance training regimen (*d* = 0.98–1.41, *p* = 0.07, strong). These results, though inconclusive, suggest that MIPS supplementation may support training-specific adaptations but does not result in systemic increases in muscle strength.

Several unique ingredients not common to all MIPS warrant mention. Kreipke et al. [59] found that resistance-trained participants who consumed a supplement containing long jack root had greater gains in bench press strength relative to placebo (*d* = 0.05, *p* = 0.001, very weak). The treatment group also improved deadlift 1RM relative to body mass and lean mass. Another group utilizing a product that has since been discontinued due to containing traces of a methamphetamine derivative [3] found no benefit of added betaine, citrulline, or *dendrobium* extract compared to placebo on strength-related outcomes. Likewise, Jung and associates [60] found no effect of chronic consumption of a MIPS containing *Mucana pruitiens* extract (15% L-Dopa) or the same supplement with the addition of synephrine on 1RM bench press when compared to placebo.

#### Muscular endurance

Limited research has examined the effect of chronic MIPS ingestion on muscular endurance, and the results are challenging to interpret as some studies are comparator controlled, while others are placebo controlled. Several studies conducted in resistance-trained participants utilizing whole-body training regimens reported beneficial results of MIPS relative to a comparator or placebo [51, 58]. Schmitz and colleagues [51] found that subjects who consumed MIPS containing a proprietary blend of ingredients performed more bodyweight bench press RTF compared to those who consumed a creatine, carbohydrate and protein-matched comparator (RE = 131.8, *p* = 0.004). Similarly, results reported by Willems et al. [58] suggest that repetitions to fatigue were enhanced at 80% pre-intervention 1-RM in participants who consumed a MIPS relative to those who consumed placebo (*d* = 1.2–1.3, *p* < 0.05, strong). Conversely, several studies found no effect of long-term MIPS consumption on muscular endurance. Spillane et al. [61] investigated the effect of the same supplement used by Schmitz et al. [51] and found no differences in RTF between those who consumed the experimental supplement relative to a comparator product. However, these differences may be due in part to differences between training interventions, as the program implemented by Spillane and colleagues [61] was shorter (6 weeks vs. 9 weeks) than that used in the experiment by Schmitz et al. [51]. Similarly, Hoffman et al. [36] found no effect of chronic MIPS supplementation on maximum pushups or sit-ups completed during a minute.

#### Power production

Preliminary evidence suggests that long-term MIPS supplementation may not augment lower-body power production. Ormsbee et al. [55] reported that trained males consuming both MIPS and placebo who participated in resistance training 3 days per week for 6 weeks experienced significant improvements in peak anaerobic power during a Wingate test. Post hoc testing identified MIPS users as having significantly increased their peak power while placebo users remained unchanged. However, these results must be interpreted with caution, as no statistically significant interaction (i.e. group x time) was identified (*p* > 0.05). Hoffman and colleagues [36] likewise found no effect of 4 weeks of MIPS supplementation on either peak or mean power measured during a Wingate test. Though evidence seems to indicate that MIPS does not affect power production, more research is needed to verify this hypothesis.

#### Endurance exercise performance

Limited research exists investigating aerobic performance following long-term MIPS ingestion, and results of these studies are contradictory. Stout and colleagues [62] found that peak oxygen consumption (VO_2peak_) and time to exhaustion during a graded cycling exercise test significantly increased by 8.4% and 5.7%, respectively, among sedentary men and women who consumed a MIPS containing caffeine, green tea extract, and glucuronolactone but not in those who consumed placebo during a 10-week resistance and aerobic exercise regimen (NA). However, Smith et al. [63] found no between-group differences in maximal oxygen consumption (VO_2max_) after 3 weeks of HIIT training in recreationally-trained males who consumed MIPS or placebo. Surprisingly, Kendall et al. [57] found that 4 weeks of supplementation with a MIPS containing caffeine, creatine, and β-alanine in recreationally-trained males resulted in decreased relative VO_2max_, though no such change was found in those who consumed a placebo (*d* = − 0.51, *p* < 0.05, moderate). However, the percent of VO_2max_ at which ventilatory threshold occurred significantly increased in the treatment but not placebo group (*d* = 1.21, *p* < 0.05, strong). The contrasting results offered by Kendall and colleagues may be explained by the lack of a specific training intervention, as all participants reported physical activity levels via questionnaire. However, given the body of research it appears that long-term MIPS consumption may augment aerobic performance in sedentary individuals beginning an exercise program or among aerobically fit individuals beginning a high-intensity protocol. However, more research is needed in this area.

#### Muscle damage

Early evidence suggests that long-term MIPS consumption has little effect on soreness, inflammation, and biomarkers of muscle damage resulting from exercise. Ormsbee et al. [56] found no effect of 4 weeks of MIPS consumption on perceived soreness, post-damage muscle function, or biochemical measures of muscle damage in endurance-trained males who performed a damaging downhill running bout. These results were mirrored in a trained female population by Köhne et al. [64], who reported that participants who consumed MIPS for 28 days prior to and 3 days after a bout of downhill running had no significant changes in soreness, muscle function, biomarkers of muscle damage, or inflammation, though effect size analysis suggested that MIPS slightly attenuated the inflammatory response to exercise. Clearly, more information is required, though initial results do not appear promising.

#### Body composition

The effect of MIPS on body composition appears to be promising, though differences in study design and the reliance on self-reported dietary intake make conclusions challenging. Several studies have found that the long-term consumption of MIPS in resistance trained males leads to greater increases in fat-free mass (FFM) following six [55, 65] (*d* = 0.18–0.22, *p* < 0.05, weak) or 8 weeks [52] (RE = 104.1, *p* < 0.01) of resistance training (three sessions per week) compared to a placebo. While the aforementioned investigations used the same brand of MIPS, participants also consumed a protein-based post-workout supplement during each of the six-week interventions, thus potentially confounding the conclusions regarding the isolated effect of MIPS supplementation that can be drawn from these studies. Chronic MIPS consumption has also been shown to improve body composition in untrained populations, as Shelmadine et al. [53] (*d* = 0.24, *p* = 0.001, weak) and Spillane et al. [54] (*d* = 0.17, *p* = 0.023, weak) noted greater gains in FFM after 28 days of resistance training in non-resistance trained males consuming MIPS compared to placebo. Additionally, Spillane and colleagues found a significantly greater decrease in fat mass (FM) in the treatment group (*d* = − 0.08, *p* = 0.026, very weak), though these effects may be due in part to post-exercise protein ingestion in addition to any chronic effect of MIPS supplementation. Stout and coworkers [62] examined the effect of MIPS ingestion on body composition in sedentary men and women during a 10-week resistance and endurance training program and found that MIPS users lost significantly more FM (NA) relative to placebo users at the end of the intervention. Interestingly, the MIPS formulation employed in this investigation was the only supplement to result in positive changes in body composition that did not contain creatine.

Few placebo-controlled studies have found MIPS to have no influence on body composition. Jung et al. [60] examined the effects of a MIPS containing *Mucana pruitiens* extract (15% L-Dopa), an identical MIPS with added synephrine, or a placebo in 80 resistance-trained males and found no effect of either MIPS formulation on body composition relative to placebo after four or 8 weeks of training. Smith et al. [63] also found no differences in body composition between MIPS and placebo users after 3 weeks of high-intensity interval training. Similarly, one of the only studies to exclusively use female subjects [66] found no change in body composition in females who consumed MIPS while participating in 7 weeks of resistance training. Thus, it seems that long-term supplementation of MIPS that contain creatine, β-alanine, whey protein, or BCAAs may exert a favorable effect on body composition in males participating in resistance training.

Several comparator-controlled studies have investigated the effect of long-term MIPS consumption on body composition, with contrasting results. Kedia et al. [3] and Spillane et al. [61] both found no improvements in body composition after 6 weeks of resistance training and supplementation among resistance-trained individuals assigned to consume MIPS as opposed to comparator product. However, another study reported significantly increased FFM (RE = 102.1, *p* = 0.049) and decreased FM (RE = 87.1, *p* = 0.023) after 9 weeks of resistance training in resistance trained males who consumed a MIPS with a proprietary blend of amino acids compared to subjects who consumed a carbohydrate, whey, and creatine-matched comparator [51]. It is important to note that the MIPS formulation used by Kedia and colleagues [3] was primarily focused on promoting energy, with the comparator matched for energy and caffeine, while the comparator products used by Spillane et al. [61] and Schmitz et al. [51] were matched for carbohydrate, whey, and creatine. The results of these studies support the idea that creatine and protein are likely the primary ingredients driving increases in fat-free mass accretion.

#### Skeletal muscle adaptations

Three studies have utilized muscle biopsy techniques to identify changes in skeletal muscle after extended exposure to MIPS. Shelmadine and colleagues [53] found MIPS to increase myofibrillar protein content (*d* = 1.78, *p* = 0.014, strong), total DNA (*d* = 2.96, *p* = 0.041, strong), as well as myogenic regulatory factors Myo-D (NA) and MRF-4 (NA) relative to placebo. Similarly, Spillane et al. [54] found MIPS to increase myofibrillar protein content (*d* = 0.32, *p* = 0.049, weak) as well as Myo-D (*d* = 0.51, *p* = 0.038, moderate) and MRF-4 (*d* = 1.37, *p* = 0.001, strong). In a later study, the same research group observed increased total muscle creatine and total muscle protein in both MIPS users and those who consumed a protein, carbohydrate, and creatine-matched comparator during 6 weeks of heavy resistance training [61]. In summary, as all of these experiments found beneficial effects of long-term supplementation of MIPS that contained whey protein, creatine, and BCAAs, these ingredients likely lead to favorable skeletal muscle adaptations.

#### Subjective measures

Several long-term MIPS studies have utilized the profile of mood states (POMS) assessment to measure psychological responses to MIPS exposure. Ormsbee et al. [65] investigated the subjective effects of MIPS consumption and found no between-groups differences in mood state, as training led to increased vigor in both those who consumed MIPS or placebo. Similarly, Kreipke and colleagues [59] did not witness any between-group differences in mood state, as an increase in anger was found in both participants who consumed placebo and participants who consumed a MIPS containing long jack root. Kedia and coworkers [3] documented the only difference in subjective measures between MIPS and comparator users and reported that MIPS led to increased energy and focus during endurance testing as well as significantly higher self-perceived energy, concentration, and focus (NA). The MIPS group also reported reductions in self-perceived fatigue. However, the product utilized in this experiment was later recalled for containing methamphetamine analogs, which may explain many of the alterations in subjective measures. Hoffman et al. [36] likewise reported that recreationally-active subjects who consumed MIPS for 4 weeks were able to maintain focus after a bout of exhaustive exercise, while those who consumed placebo experienced declines in focus (NA).

#### Reaction time and cognitive processing

It appears that chronic MIPS supplementation has little effect on reaction time, as Hoffman and colleagues [36] found no effect of a caffeine-containing supplement on reaction time after a bout of exhaustive exercise. Jung et al. [60] found that those who consumed a MIPS containing caffeine, L-tyrosine, and L-Dopa (derived from *Mucana puritens* extract) with or without synephrine had better performance during a Stroop test (*d* = 0.12–0.16, *p* < 0.05, very weak). However, as only a single study using a unique ingredient has investigated cognitive functioning after more than 10 days of MIPS exposure, more research is needed to bolster any conclusion drawn from these findings.

#### Hormonal response

The hormonal response to long-term MIPS use has only been evaluated in resistance trained males. In several resistance-training studies which used the same MIPS formulation, no changes were found in cortisol [65], GH [55], or insulin-like growth factor-1 (IGF-1) [55] relative to placebo. A similar product containing whey, creatine, BCAAs, and taurine also had little effect on insulin, IGF-1, cortisol, or GH relative to placebo [61]. While testosterone has been shown to increase following training among both MIPS and placebo users [55], no study has yet demonstrated an effect of chronic MIPS supplementation on testosterone concentration relative to placebo. Furthermore, consumption of a MIPS containing long jack root had no effect on bioavailable testosterone, free testosterone, total testosterone, sex-hormone binding globulin, or estradiol compared to placebo [59].

#### Gender

Four long-term supplementation studies included in this review examined the effects of MIPS on both males and females, while an additional two investigations only examined female participants. Stout et al. [62] included 20 males and 18 females in their study but did not control for menstrual cycle or assess the effect of gender on any outcome variable. Similarly, Kedia and colleagues [3] enrolled men and women into their 6-week long study, though the authors did not disclose the ratio of females to males or assess gender as a covariate. However, the researchers controlled for the menstrual cycle. The sample obtained by Hoffman et al. [36] was only 11% female (2/19 total participants) and researchers did not control for menstrual cycle or include gender in their analysis, likely due to the discrepancies in sample size. Köhne and colleagues [64] explored the effects of a MIPS on flexibility, muscle damage, and power in female runners who performed a single bout of downhill running during the mid-follicular phase following 28 days of supplementation. Though Köhne and colleagues did not find any differences between MIPS and placebo with regard to any outcome measure, these results are very similar to a study performed in men using a nearly identical study design and the same supplement [56]. Another study using only female participants found that MIPS did not influence RMR or body composition when compared to placebo [66], though menstrual control was not mentioned in the manuscript. No consensus can be drawn from these data on the effect of gender on long-term MIPS consumption. Future studies should simultaneously compare men and women following the same training protocol and using the same supplement while controlling for hormonal status in female participants.

## Safety implications of MIPS use

To date, relevant literature suggests that the consumption of many MIPS appears to be relatively safe with minimal reported adverse effects. However, most studies examining the effects of MIPS ingestion are relatively short (less than 8 weeks). Several studies have examined the effects of chronic MIPS ingestion on heart rate, blood pressure and several hematologic markers with minimal adverse effects reported [3, 53, 57, 67]. These effects of varying durations of MIPS use on health-related outcomes are outlined below in their respective sections. It is vital to note that many studies only report mean changes within the entire sample and do not specify whether a few individuals exceeded normal ranges at any point during the testing period. This reporting technique may mask adverse events if individual changes above normal ranges are not clearly reported in a manner similar to that employed by Jung and colleagues [60]. Future investigations should employ this approach to present a more comprehensive picture of the effects of MIPS on various clinical markers of safety. With that being said, as with many prescription medications, short-term use often tends to be relatively safe with few serious adverse effects. However, clinical manifestations may take months or even years to become present. Therefore, longer-term data is needed to determine potential adverse physiologic adaptations due to chronic exposure to MIPS. Previous reviews by Eudy et al. [27] and Maughan et al. [68] have comprehensively outlined potential adverse effects of frequently used ingredients in various sports supplements including MIPS.

Concerns regarding safety of supplement use go far beyond merely examining the ingredient list that declared on the supplement label, as several supplements have been found to contain contaminants such as heavy metals, potent stimulants, or various banned ingredients [69–71]. Alarmingly, Geyer et al. [72] reported that up to 15% of dietary supplements contained hormones or prohormones. Similarly, Cohen [73] warned that potential contaminants ranged from mere impurities to harmful chemicals, medications, and banned substances. Furthermore, he cautioned that the dose of these contaminants may vary widely, from sub-therapeutic, but detectable, to potentially toxic doses. Potent and questionably legal stimulants or stimulant derivatives may be the most common of such culprits as they may provide additional stimulatory effects beyond those of caffeine. Furthermore, such stimulants could reinforce supplement consumption in MIPS users who may base supplement purchasing decisions on the intensity of perceived stimulatory effects following ingestion. One particularly concerning report published in 2014 [69] indicated that a methamphetamine analog was identified in a popular MIPS product, which prompted its removal from the market by request of the FDA. 1,3-dimethylamylamine (DMAA), a similar potent sympathomimetic ingredient once found in a variety of pre-workout supplements, was likewise removed from the market by the FDA after being implicated as the cause of six deaths and over 100 reported illnesses due to its detrimental effect on blood pressure and cardiac function [74]. Unsurprisingly, several unregulated DMAA analogs such as Octodrine (2-amino-6-methylheptane) and 1,4-dimethylamylamine have recently been identified in a sample of dietary supplements [75]. Due to the reactive rather than proactive nature of supplement regulations in the United States, it is likely that ever-evolving iterations of experimental stimulants will be present in a variety of products as earlier versions of these substances are regulated and likely banned.

Thus, any athlete consuming a supplement runs the risk of failing a drug test if components of the supplement are banned by their respective organization, which could lead to disqualification from participation or loss of employment. Adverse effects from consuming a supplement can result from ingestion of known ingredients and also from any unknown contaminants or inadvertent ingestion of mega doses of the listed ingredients. Many supplement labels list ingredients as “proprietary blends” leaving the consumer to merely guess how much of a given compound is in the product. This could lead to inadvertent overdose of various ingredients, particularly if they are also being consumed in other products, food items, or dietary supplements. As mentioned earlier, caffeine is a main ingredient in many MIPS and consumption of the substance in high doses, whether intentional or unintentional, can result in nausea, heart palpitations, arrhythmias, and headache [76–78]. Proper dosage of each ingredient is critical as it pertains to potential performance benefits and certainly as it pertains to adverse effects. When individual ingredients found in most supplements are used at recommended dosages, most are well tolerated. A well-respected program, Operation Supplement Safety (OPSS.org) has compiled a list of high risk supplements that could contain banned substances such as harmful stimulants, anabolic steroids or other hormones that could negatively affect one’s health. The concerned athlete should verify all supplement purchases using such a method prior to consumption to ensure that risk of contamination is minimized.

### Safety of acute MIPS consumption

#### Blood chemistry

Jung and colleagues [60], using a MIPS that was later tested in a chronic use study, found no changes to liver function after acute ingestion of a single dose. However, there was a significant group x time interaction for some measures of kidney function (blood urea nitrogen, creatinine) in participants consuming MIPS (*d* = − 0.18 - 0.52, *p* < 0.05, weak - moderate). These changes were expected due to the creatine content of the supplement and were still within normal clinical ranges. Likewise, Collins et al. [23] noted no change in liver or kidney function markers approximately 24 h after MIPS ingestion.

#### Hemodynamic response

Acute supplementation with MIPS does not appear to adversely impact hemodynamic variables, though a variety of responses are noted. Ingestion of a caffeine-based MIPS has been demonstrated to have no effect on heart rate (HR), systolic blood pressure (SBP), or diastolic blood pressure (DBP) within 30 min of ingestion [79]. Kedia et al. [3] found that ingestion of the MIPS supplement which was later banned for containing a methamphetamine analog resulted in elevations in SBP for 30–120 min post ingestion (*d* = 0.5–0.81, *p* < 0.03, moderate - strong). Another study demonstrated elevated DBP but not SBP after MIPS ingestion (*d* = 15.9, *p* = 0.011, strong) [4]. Several investigations have noted higher post-exercise HR in caffeine-based MIPS users [37, 50]. Based upon the well-documented physiological effect of the caffeine contained in these supplements, these responses are not unexpected, and no response was outside an acceptable normal range. The average time necessary for orally-ingested caffeine to reach peak plasma concentration (~ 30–60 min) likely explains the discrepancies between studies which saw no differences at 30 min versus elevations at time points greater than 30 min [80].

### Safety of short-term MIPS supplementation (< 10 days)

#### Blood chemistry

One study has investigated the safety of chronic MIPS use for a period of less than 10 days. Collins et al. [23] reported no difference in clinical serum or whole blood health markers between participants using MIPS or placebo for 7 days. Short term MIPS use does not appear to induce undesirable blood chemistry and may provide a favorable hormonal environment for those trying to perform.

#### Hemodynamic response

Using a tilt-table, Collins and colleagues [23] performed a hemodynamic challenge test wherein participants rested supine for 15 min, and were then positioned vertically for 2 minutes, with HR and blood pressure being assessed immediately before and after the 2 minutes of vertical positioning. No unexpected results were seen, and there were no significant differences between MIPS and placebo with regards to HR, SBP, DBP, mean arterial pressure (MAP), or rate pressure product (RPP).

### Safety of long-term MIPS supplementation (> 10 days)

#### Blood chemistry

Evidence suggests that long-term MIPS supplementation has a largely benign effect on blood chemistry parameters. Many studies have reported that long term MIPS use has no deleterious effects on blood lipid profile [3, 51, 54, 59, 60, 65], blood glucose [51, 54, 59, 65], cortisol [61, 65], IGF-1 [53–55, 59, 61], liver enzymes [51, 54, 57, 60], kidney function [3, 51, 54, 57], or other standard blood chemistry values obtained within a comprehensive metabolic panel [51, 53]. While Spillane et al. [54] noted a lower basophil count among MIPS users relative to placebo (*d* = − 1.06, *p* = 0.05, strong) following a 28-day training program, these changes were not outside of normal limits. Overall, these results suggest that MIPS ingestion for greater than 10 days is unlikely to negatively affect blood chemistry values.

#### Hemodynamic response

No study has found MIPS to have a different effect on hemodynamic variables relative to placebo supplementation. Ormsbee et al. [65] found no differences in RHR, SBP or DBP relative to placebo after supplementation with a whey, BCAA, creatine, β-alanine, and arginine-based MIPS with a protein post-workout supplement. Kendall et al. [57] likewise noted no differences compared to placebo in RHR, SBP, DBP after 4 weeks of supplementation with a MIPS containing caffeine, creatine, β-alanine, and amino acids. Similarly, the addition of synephrine to a MIPS containing L-Dopa had no detectable impact on RHR, SBP, or DBP relative to placebo or identical MIPS without synephrine [60]. Interestingly, Kedia and colleagues [3] also found no effect of long-term MIPS supplementation on RHR, SBP, or DBP despite supplementing with a formulation that was demonstrated to contain methamphetamine analogs. Overall, MIPS appears to be hemodynamically safe.

## Conclusions

Clearly, the body of literature and quality studies examining the efficacy and safety of MIPS supplementation is preliminary at best. In this review, we have comprehensively outlined all available empirical evidence on MIPS consumption. The majority of the current literature would suggest that supplementation with these products improves various types of exercise performance and can potentiate training adaptations. Studies that directly examined safety parameters and adverse effects of MIPS consumption concluded that short-term supplementation is safe in otherwise healthy consumers. However, we would recommend discussing specific products and dosages of any supplement with a knowledgeable health professional or sports dietician prior to ingesting any product. As stated previously, it is important to understand how different ingredients in sports supplements may interact with prescription or over the counter medications. Using a trusted verification source and third party testing can also help to minimize the possibility of ingesting potentially harmful contaminants in these products. Given the potential beneficial effects of these supplements demonstrated in the literature to date, further investigations are warranted. Anecdotally, we know that many people consume these products for many years. Therefore, further research is needed to determine the effects of chronic MIPS consumption on performance enhancement, training adaptions, and markers of health and safety. Unfortunately, until the FDA implements additional regulations that require companies to bring forth safety data in humans of durations longer than 8–12 weeks, it is unlikely studies longer than this will become available. Similarly, more information is required concerning the effects of MIPS consumption in a wider variety of under-researched populations such as female athletes as well as untrained adults above 40 years of age. Further evidence concerning the effect of MIPS supplementation on measures of sport specific performance is also needed.

## Abbreviations

1RM: One repetition maximum
ALT: Alanine aminotransferase
AST: Aspartate aminotransferase
BCAA: Branched-chain amino acid
BUN: Blood urea nitrate
*d*: Effect size (Cohen’s *d*)
DBP: Diastolic blood pressure
DMAA: 1,3-dimethylamylamine
FDA: United States Food and Drug Administration
FFM: Fat-free mass
FM: Fat mass
GH: Growth hormone
HR: Heart rate
IGF-1: Insulin-like growth factor 1
MAP: Mean arterial pressure
MIPS: Multi-ingredient pre-workout supplement
NO: Nitric Oxide
POMS: Profile of mood states
RE: Relative effects
REE: Resting energy expenditure
RER: Respiratory exchange ratio
RPP: Rate pressure product
RTF: Repetitions to fatigue
SBP: Systolic blood pressure
SD: Standard deviation
VO_2max_: Maximal oxygen consumption
VO_2peak_: Peak oxygen consumption

## References

[1] Froiland K, Koszewski W, Hingst J, Kopecky L (2004). Nutritional supplement use among college athletes and their sources of information. Int J Sport Nutr Exerc Metab..

[2] Wardenaar F, van den Dool R, Ceelen I, Witkamp R, Mensink M (2016). Self-reported use and reasons among the general population for using sports nutrition products and dietary supplements. Sports.

[3] Kedia AW, Hofheins JE, Habowski SM, Ferrando AA, Gothard MD, Lopez HL (2014). Effects of a pre-workout supplement on lean mass, muscular performance, subjective workout experience and biomarkers of safety. Int J Med Sci.

[4] Cameron M, Camic CL, Doberstein S, Erickson JL, Jagim AR (2018). The acute effects of a multi-ingredient pre-workout supplement on resting energy expenditure and exercise performance in recreationally active females. J Int Soc Sports Nutr..

[5] Jagim AR, Jones MT, Wright GA, St Antoine C, Kovacs A, Oliver JM (2016). The acute effects of multi-ingredient pre-workout ingestion on strength performance, lower body power, and anaerobic capacity. J Int Soc Sports Nutr.

[6] Lane M, Byrd M (2018). Effects of pre-workout supplements on power maintenance in lower body and upper body tasks. J Funct Morphol Kinesiol.

[7] Shields KA, Silva JE, Rauch JT, Lowery RP, Ormes JA, Sharp MH, McCleary SA, Georges J, Joy JM, Purpura M (2014). The effects of a multi-ingredient cognitive formula on alertness, focus, motivation, calmness and psychomotor performance in comparison to caffeine and placebo. J Int Soc Sports Nutr..

[8] Faraone SV (2008). Interpreting estimates of treatment effects: implications for managed care. Pharm Ther.

[9] Trexler ET, Smith-Ryan AE, Stout JR, Hoffman JR, Wilborn CD, Sale C, Kreider RB, Jäger R, Earnest CP, Bannock L (2015). International society of sports nutrition position stand: Beta-alanine. J Int Soc Sports Nutr..

[10] Campbell B, Wilborn C, La Bounty P, Taylor L, Nelson MT, Greenwood M, Ziegenfuss TN, Lopez HL, Hoffman JR, Stout JR (2013). International Society of Sports Nutrition position stand: energy drinks. J Int Soc Sports Nutr..

[11] Goldstein ER, Ziegenfuss T, Kalman D, Kreider R, Campbell B, Wilborn C, Taylor L, Willoughby D, Stout J, Graves BS (2010). International society of sports nutrition position stand: caffeine and performance. J Int Soc Sports Nutr..

[12] Spriet LL (2014). Exercise and sport performance with low doses of caffeine. Sports Med.

[13] Graham TE (2001). Caffeine and exercise: metabolism, endurance and performance. Sports Med.

[14] Fredholm BB, Battig K, Holmen J, Nehlig A, Zvartau EE (1999). Actions of caffeine in the brain with special reference to factors that contribute to its widespread use. Pharmacol Rev.

[15] Galloway SDR, Talanian JL, Shoveller AK, Heigenhauser GJF, Spriet LL (2008). Seven days of oral taurine supplementation does not increase muscle taurine content or alter substrate metabolism during prolonged exercise in humans. J Appl Physiol.

[16] Hoffman JR, Ratamess NA, Ross R, Shanklin M, Kang J, Faigenbaum AD (2008). Effect of a pre-exercise energy supplement on the acute hormonal response to resistance exercise. J Strength Cond Res..

[17] Wolfe RR (2017). Branched-chain amino acids and muscle protein synthesis in humans: myth or reality?. J Int Soc Sports Nutr..

[18] Campbell B, Kreider RB, Ziegenfuss T, La Bounty P, Roberts M, Burke D, Landis J, Lopez H, Antonio J (2007). International Society of Sports Nutrition position stand: protein and exercise. J Int Soc Sports Nutr..

[19] Domínguez R, Cuenca E, Maté-Muñoz JL, García-Fernández P, Serra-Paya N, Estevan MCL, Herreros PV, Garnacho-Castaño MV (2017). Effects of beetroot juice supplementation on cardiorespiratory endurance in athletes. A Systematic Review Nutrients.

[20] Domínguez R, Maté-Muñoz JL, Cuenca E, García-Fernández P, Mata-Ordoñez F, Lozano-Estevan MC, Veiga-Herreros P, da Silva SF, Garnacho-Castaño MV (2018). Effects of beetroot juice supplementation on intermittent high-intensity exercise efforts. J Int Soc Sports Nutr.

[21] Clements WT, Lee S-R, Bloomer RJ (2014). Nitrate ingestion: a review of the health and physical performance effects. Nutrients.

[22] Bescos R, Sureda A, Tur JA, Pons A (2012). The effect of nitric-oxide-related supplements on human performance. Sports Med.

[23] Collins PB, Earnest CP, Dalton RL, Sowinski RJ, Grubic TJ, Favot CJ, Coletta AM, Rasmussen C, Greenwood M, Kreider RB. Short-erm effects of a ready-to-drink pre-workout beverage on exercise performance and recovery. Nutrients. 2017;9(8):823. 10.3390/nu9080823 PMC5579616 28763003

[24] Alvares TS, Meirelles CM, Bhambhani YN, Paschoalin VM, Gomes PS (2011). L-arginine as a potential ergogenic aid in healthy subjects. Sports Med.

[25] Figueroa A, Wong A, Jaime SJ, Gonzales JU (2017). Influence of L-citrulline and watermelon supplementation on vascular function and exercise performance. Curr Opin Clin Nutr Metab Care.

[26] Kreider RB, Kalman DS, Antonio J, Ziegenfuss TN, Wildman R, Collins R, Candow DG, Kleiner SM, Almada AL, Lopez HL (2017). International Society of Sports Nutrition position stand: safety and efficacy of creatine supplementation in exercise, sport, and medicine. J Int Soc Sports Nutr..

[27] Eudy AE, Gordon LL, Hockaday BC, Lee DA, Lee V, Luu D, Martinez CA, Ambrose PJ (2013). Efficacy and safety of ingredients found in preworkout supplements. Am J Health Syst Pharm.

[28] Antonio J, Ciccone V (2013). The effects of pre versus post workout supplementation of creatine monohydrate on body composition and strength. J Int Soc Sports Nutr..

[29] Hoffman JR, Ratamess NA, Kang J, Gonzalez AM, Beller NA, Craig SAS (2011). Effect of 15 days of betaine ingestion on concentric and eccentric force outputs during isokinetic exercise. J Strength Cond Res..

[30] Trepanowski JF, Farney TM, McCarthy CG, Schilling BK, Craig SA, Bloomer RJ (2011). The effects of chronic betaine supplementation on exercise performance, skeletal muscle oxygen saturation and associated biochemical parameters in resistance trained men. J Strength Cond Res.

[31] Lee EC, Maresh CM, Kraemer WJ, Yamamoto LM, Hatfield DL, Bailey BL, Armstrong LE, Volek JS, McDermott BP, Craig SAS (2010). Ergogenic effects of betaine supplementation on strength and power performance. J Int Soc Sports Nutr..

[32] Tinsley GM, Hamm MA, Hurtado AK, Cross AG, Pineda JG, Martin AY, Uribe VA, Palmer TB (2017). Effects of two pre-workout supplements on concentric and eccentric force production during lower body resistance exercise in males and females: a counterbalanced, double-blind, placebo-controlled trial. J Int Soc Sports Nutr.

[33] Bergstrom HC, Byrd MT, Wallace BJ, Clasey JL (2018). Examination of A Multi-Ingredient Pre-Workout Supplement on Total Volume of Resistance Exercise and Subsequent Strength and Power Performance. J Strength Cond Res.

[34] Magrini MA, Colquhoun RJ, Dawes JJ, Smith DB (2016). Effects of a pre-workout energy drink supplement on upper body muscular endurance performance. Int J Exerc Sci.

[35] Bloomer RJ, Farney TM, Trepanowski JF, McCarthy CG, Canale RE, Schilling BK (2010). Comparison of pre-workout nitric oxide stimulating dietary supplements on skeletal muscle oxygen saturation, blood nitrate/nitrite, lipid peroxidation, and upper body exercise performance in resistance trained men. J Int Soc Sports Nutr..

[36] Hoffman JR, Ratamess NA, Gonzalez A, Beller NA, Hoffman MW, Olson M, Purpura M, Jäger R (2010). The effects of acute and prolonged CRAM supplementation on reaction time and subjective measures of focus and alertness in healthy college students. J Int Soc Sports Nutr..

[37] Spradley BD, Crowley KR, Tai CY, Kendall KL, Fukuda DH, Esposito EN, Moon SE, Moon JR (2012). Ingesting a pre-workout supplement containing caffeine, B-vitamins, amino acids, creatine, and beta-alanine before exercise delays fatigue while improving reaction time and muscular endurance. Nutr Metab (Lond).

[38] Gonzalez AM, Walsh AL, Ratamess NA, Kang J, Hoffman JR (2011). Effect of a pre-workout energy supplement on acute multi-joint resistance exercise. J Sports Sci Med.

[39] Jung YP, Earnest CP, Koozehchian M, Galvan E, Dalton R, Walker D, Rasmussen C, Murano PS, Greenwood M, Kreider RB (2017). Effects of acute ingestion of a pre-workout dietary supplement with and without p-synephrine on resting energy expenditure, cognitive function and exercise performance. J Int Soc Sports Nutr..

[40] Jacobs PL (2014). The acute effects of a commercial pre workout product, wodFuel®, on performance of a Crossfit exercise series, the Cindy. J Int Soc Sports Nutr..

[41] Martinez N, Campbell B, Franek M, Buchanan L, Colquhoun R (2016). The effect of acute pre-workout supplementation on power and strength performance. J Int Soc Sports Nutr..

[42] Hahn CJ, Jagim AR, Camic CL, Andre MJ (2018). The acute effects of a caffeine-containing supplement on anaerobic power and subjective measurements of fatigue in recreationally-active males. J Strength Cond Res..

[43] Hoffman JR, Kang J, Ratamess NA, Hoffman MW, Tranchina CP, Faigenbaum AD (2009). Examination of a pre-exercise, high energy supplement on exercise performance. J Int Soc Sports Nutr..

[44] Walsh AL, Gonzalez AM, Ratamess NA, Kang J, Hoffman JR (2010). Improved time to exhaustion following ingestion of the energy drink amino impact. J Int Soc Sports Nutr.

[45] Musgjerd T, Johnston N, Jagim A, Camic C (2018). Acute effects of a multi-ingredient pre-workout supplement on 5-km running performance in recreationally-trained athletes. Med Sci Sports Exerc.

[46] Ratamess NA, Hoffman JR, Ross R, Shanklin M, Faigenbaum AD, Kang J (2007). Effects of an amino acid/creatine energy supplement on the acute hormonal response to resistance exercise. Int J Sport Nutr Exerc Metab.

[47] Erickson J, Jagim A, Wright G, Foster C, Camic C (2018). Effects of a thermogenic pre-workout supplement on fat oxidation rates during moderate-intensity running in females. Med Sci Sports Exerc.

[48] Outlaw JJ, Wilborn CD, Smith-Ryan AE, Hayward SE, Urbina SL, Taylor LW, Foster CA (2014). Acute effects of a commercially-available pre-workout supplement on markers of training: a double-blind study. J Int Soc Sports Nutr.

[49] Kraemer WJ, Hatfield DL, Spiering BA, Vingren JL, Fragala MS, Ho J-Y, Volek JS, Anderson JM, Maresh CM (2007). Effects of a multi-nutrient supplement on exercise performance and hormonal responses to resistance exercise. Eur J Appl Phys.

[50] Martin JS, Mumford PW, Haun CT, Luera MJ, Muddle TWD, Colquhoun RJ, Feeney MP, Mackey CS, Roberson PA, Young KC (2017). Effects of a pre-workout supplement on hyperemia following leg extension resistance exercise to failure with different resistance loads. J Int Soc Sports Nutr.

[51] Schmitz SM, Hofheins JE, Lemieux R. Nine weeks of supplementation with a multi-nutrient product augments gains in lean mass, strength, and muscular performance in resistance trained men. J Int Soc Sports Nutr. 2010;7:40. 10.1186/1550-2783-7-40 PMC3016253 21162744

[52] Lowery RP, Joy JM, Dudeck JE, Oliveira de Souza E, SA MC, Wells S, Wildman R, Wilson JM (2013). Effects of 8 weeks of Xpand(R) 2X pre workout supplementation on skeletal muscle hypertrophy, lean body mass, and strength in resistance trained males. J Int Soc Sports Nutr.

[53] Shelmadine B, Cooke M, Buford T, Hudson G, Redd L, Leutholtz B, Willoughby DS (2009). Effects of 28 days of resistance exercise and consuming a commercially available pre-workout supplement, NO-shotgun(R), on body composition, muscle strength and mass, markers of satellite cell activation, and clinical safety markers in males. J Int Soc Sports Nutr..

[54] Spillane M, Schwarz N, Leddy S, Correa T, Minter M, Longoria V, Willoughby DS (2011). Effects of 28 days of resistance exercise while consuming commercially available pre- and post-workout supplements, NO-Shotgun(R) and NO-Synthesize(R) on body composition, muscle strength and mass, markers of protein synthesis, and clinical safety markers in males. Nutr Metab (Lond).

[55] Ormsbee MJ, Mandler WK, Thomas DD, Ward EG, Kinsey AW, Simonavice E, Panton LB, Kim JS (2012). The effects of six weeks of supplementation with multi-ingredient performance supplements and resistance training on anabolic hormones, body composition, strength, and power in resistance-trained men. J Int Soc Sports Nutr..

[56] Ormsbee MJ, Ward EG, Bach CW, Arciero PJ, McKune AJ, Panton LB (2015). The impact of a pre-loaded multi-ingredient performance supplement on muscle soreness and performance following downhill running. J Int Soc Sports Nutr.

[57] Kendall KL, Moon JR, Fairman CM, Spradley BD, Tai CY, Falcone PH, Carson LR, Mosman MM, Joy JM, Kim MP (2014). Ingesting a preworkout supplement containing caffeine, creatine, beta-alanine, amino acids, and B vitamins for 28 days is both safe and efficacious in recreationally active men. Nutr Res.

[58] Willems ME, Sallis CW, Haskell JA (2012). Effects of multi-ingredient supplementation on resistance training in young males. J Hum Kinet.

[59] Kreipke VC, Allman BR, Kinsey AW, Moffatt RJ, Hickner RC, Ormsbee MJ (2015). Impact of four weeks of a multi-ingredient performance supplement on muscular strength, body composition, and anabolic hormones in resistance-trained young men. J Strength Cond Res..

[60] Jung YP, Earnest CP, Koozehchian M, Cho M, Barringer N, Walker D, Rasmussen C, Greenwood M, Murano PS, Kreider RB (2017). Effects of ingesting a pre-workout dietary supplement with and without synephrine for 8 weeks on training adaptations in resistance-trained males. J Int Soc Sports Nutr..

[61] Spillane M, Schwarz N, Willoughby DS (2014). Heavy resistance training and Peri-exercise ingestion of a multi-ingredient ergogenic nutritional supplement in males: effects on body composition, muscle performance and markers of muscle protein synthesis. J Sports Sci Med..

[62] Stout JR, Moon JR, Tobkin SE, Lockwood CM, Smith AE, Graef JL, Kendall KL, Beck TW, Cramer JT. Pre-workout consumption of Celsius® enhances the benefits of chronic exercise on body composition and cardiorespiratory fitness. J Int Soc Sports Nutr. 2008;5(Suppl1):P8.

[63] Smith AE, Fukuda DH, Kendall KL, Stout JR (2010). The effects of a pre-workout supplement containing caffeine, creatine, and amino acids during three weeks of high-intensity exercise on aerobic and anaerobic performance. J Int Soc Sports Nutr..

[64] Köhne JL, Ormsbee MJ, McKune AJ (2016). The effects of a multi-ingredient supplement on markers of muscle damage and inflammation following downhill running in females. J Int Soc Sports Nutr.

[65] Ormsbee MJ, Thomas DD, Mandler WK, Ward EG, Kinsey AW, Panton LB, Scheett TP, Hooshmand S, Simonavice E, Kim JS (2013). The effects of pre- and post-exercise consumption of multi-ingredient performance supplements on cardiovascular health and body fat in trained men after six weeks of resistance training: a stratified, randomized, double-blind study. Nutr Metab (Lond).

[66] Zabriskie H, Camic CL, Foster C, Nelson A, Zajac B, Hoecherl K, Luedke J, Erickson J, Jagim AR. Supplementation with a multi-ingredient pre-workout supplement does not enhance body composition or metabolism in females. Int Jour Exer Sci. 2017;11(5) 10.70252/ISGV4343 PMC6355134 30761195

[67] Vogel RM, Joy JM, Falcone PH, Mosman MM, Kim MP, Moon JR (2015). Safety of a dose-escalated pre-workout supplement in recreationally active females. J Int Soc Sports Nutr..

[68] Maughan RJ, King DS, Lea T (2004). Dietary supplements. J Sports Sci.

[69] Cohen PA, Travis JC, Venhuis BJ (2014). A methamphetamine analog (N,alpha-diethyl-phenylethylamine) identified in a mainstream dietary supplement. Drug Test Anal.

[70] Maughan RJ (2012). Quality assurance issues in the use of dietary supplements, with special reference to protein supplements, 2. J Nutr.

[71] Martinez-Sanz JM, Sospedra I, Ortiz CM, Baladia E, Gil-Izquierdo A, Ortiz-Moncada R. Intended or unintended doping? A review of the presence of doping substances in dietary supplements used in sports. Nutrients. 2017;9(10):1093. 10.3390/nu9101093 PMC5691710 28976928

[72] Geyer H, Parr MK, Mareck U, Reinhart U, Schrader Y, Schanzer W (2004). Analysis of non-hormonal nutritional supplements for anabolic-androgenic steroids - results of an international study. Int J Sports Med.

[73] Cohen PA (2009). American roulette — contaminated dietary supplements. N Engl J Med.

[74] Lieberman HR, Austin KG, Farina EK (2018). Surveillance of the armed forces as a sentinel system for detecting adverse effects of dietary supplements in the general population. Public Health Nutr.

[75] Cohen PA, Travis JC, Keizers PHJ, Deuster P, Venhuis BJ. Four experimental stimulants found in sports and weight loss supplements: 2-amino-6-methylheptane (octodrine), 1,4-dimethylamylamine (1,4-DMAA), 1,3-dimethylamylamine (1,3-DMAA) and 1,3-dimethylbutylamine (1,3-DMBA). Clin Toxicol (Phila). 2018;56(6):421-26. 10.1080/15563650.2017.1398328 29115866

[76] Kaplan GB, Greenblatt DJ, Ehrenberg BL, Goddard JE, Cotreau MM, Harmatz JS, Shader RI (1997). Dose-dependent pharmacokinetics and psychomotor effects of caffeine in humans. J Clin Pharmacol.

[77] Chou T (1992). Wake up and smell the coffee. Caffeine, coffee, and the medical consequences. West J Med.

[78] Nehlig A, Daval J-L, Debry G (1992). Caffeine and the central nervous system: mechanisms of action, biochemical, metabolic and psychostimulant effects. Brain Res Rev.

[79] Baetge C, Earnest CP, Lockard B, Coletta AM, Galvan E, Rasmussen C, Levers K, Simbo SY, Jung YP, Koozehchian M (2017). Efficacy of a randomized trial examining commercial weight loss programs and exercise on metabolic syndrome in overweight and obese women. Appl Physiol Nutr Metab.

[80] Arnaud MJ (1987). The pharmacology of caffeine. Prog Drug Res.

